# Ebastine exerts antitumor activity and induces autophagy by activating AMPK/ULK1 signaling in an IPMK-dependent manner in osteosarcoma

**DOI:** 10.7150/ijbs.69541

**Published:** 2023-01-01

**Authors:** Zhen Pan, Shi-jie Li, Hua Guo, Zhao-hui Li, Xiang Fei, Shi-min Chang, Qing-cheng Yang, Dong-dong Cheng

**Affiliations:** 1 Department of Orthopedics, Shanghai Jiao Tong University Affiliated Sixth People's Hospital, Shanghai, 200233, China.; 2 Department of Orthopaedic Surgery, Yangpu Hospital, Tongji University School of Medicine, Shanghai, 200090, China.

**Keywords:** Ebastine, osteosarcoma, autophagy, IPMK, AMPK/ULK1 pathway

## Abstract

Numerous studies have confirmed that in addition to interfering with the tumor inflammatory environment, anti-inflammatory agents can directly increase apoptosis and sensitivity to conventional therapies and decrease invasion and metastasis, making them useful candidates for cancer therapy. Here, we first used high-throughput screening and had screened one compound candidate, ebastine (a H1-histamine receptor antagonist), for osteosarcoma therapy. Cell viability assays, colony formation assays, wound healing assays, and Transwell assays demonstrated that ebastine elicited antitumor effects in osteosarcoma cells. In addition, ebastine treatment exerted obvious effects on cell cycle arrest, metastasis inhibition, apoptosis and autophagy induction both *in vitro* and* in vivo*. Mechanistically, we observed that ebastine treatment triggered proapoptotic autophagy by activating AMPK/ULK1 signaling in osteosarcoma cells. Treatment with the AMPK inhibitor dorsomorphin reversed ebastine-induced apoptosis and autophagy. More importantly, we found that IPMK interacted with AMPK and functioned as a positive regulator of AMPK protein in osteosarcoma cells. A rescue study showed that the induction of autophagy and activation of the AMPK/ULK1 signaling pathway by ebastine treatment were reversed by IPMK knockdown, indicating that the activity of ebastine was IPMK dependent. We provide experimental evidence demonstrating that ebastine has antitumor activity in osteosarcoma and promotes autophagy by activating the AMPK/ULK1 signaling pathway, which is IPMK dependent. Our results provide insight into the clinical application potential of ebastine, which may represent a new potential therapeutic candidate for the treatment of osteosarcoma.

## Introduction

Osteosarcoma is a rare bone-forming malignant tumor that predominantly affects children and adolescents. The metaphysis regions of long bones, the most active sites in bone, are major sites of osteosarcoma growth 1-2. While only accounting for approximately 5% of childhood and adolescent cancers, it has a substantial impact on pediatric cancer mortality 3-4. Standard treatment for osteosarcoma consists of surgery and chemotherapy. The advent of chemotherapy in the 20th century allowed for the implementation of neoadjuvant and adjuvant chemotherapy 5-6. This improved overall survival from 20% to approximately 70% in osteosarcoma patients 7-9. However, this did not improve the outcome in patients harboring macrometastases at diagnosis or those with relapsed disease 1, 10. Osteosarcoma is a particularly chemotherapy-resistant tumor that only responds to high doses of chemotherapy and rapidly acquires resistance 11-12. The implementation of neoadjuvant and adjuvant chemotherapy has greatly increased the survival rates of osteosarcoma, but side effects can affect the quality of life of long-term survivors of childhood cancer. In addition, a plateau has been reached, and further therapy intensification has failed to improve outcomes since the 1980s 13. Therefore, identification of novel targets and the development of more effective therapeutic strategies for osteosarcoma are urgently needed.

The advent of immunotherapy has breakthrough significance for the treatment of specific cancers 14. Some studies have reported the effects of immunoregulatory agents on osteosarcoma 15-17. Survival in osteosarcoma is directly related to the level of CD8+ T cell infiltration in patient samples, which indicates that targeted immune checkpoints may be an effective strategy against osteosarcoma 18-19. Reports have suggested that human interferon can be used as an adjuvant therapy to osteosarcoma patients 20-21. IFN-α-2b regulates the immune system and exerts antiproliferative and proapoptotic effects in several cancer models, including osteosarcoma, making it a promising treatment method 22. In addition to interfering with the tumor immunomicroenviroment, anti-inflammatory agents directly increase apoptosis and sensitivity to conventional therapies and decrease invasion and metastasis, making them useful candidates for cancer therapy. Celecoxib was reported to activate denovo sphingolipid biosynthesis and induce endoplasmic reticulum (ER) stress; the combined effects of elevated ceramide and transcriptional activation of ER stress induce apoptosis in HepG2 hepatoma cells 23. Megan et al*.* found that dexamethasone induced cell death in multiple myeloma (MM), and pharmacological manipulations showed that the key regulation enabling complete dexamethasone sensitivity in MM cells lies with miR-125b 24. Eriodictyol, a natural anti-inflammatory flavonoid, has been demonstrated to inhibit glioblastoma migration and invasion by reversing EMT via downregulation of the P38 MAPK/GSK-3β/ZEB1 pathway 25.

In this study, we focused on the direct antitumor activity of anti-inflammatory agents in osteosarcoma. Thus, we performed high-throughput small molecule screening with a panel of bioactive anti-inflammatory compounds to explore drug sensitivity to osteosarcoma. Here, we demonstrated that the H1-histamine receptor antagonist compound ebastine exerted a strong antitumor effect in osteosarcoma. The findings presented here highlight the potential of inducing autophagy and apoptosis and suppressing osteosarcoma when ebastine was used.

## Materials and methods

### Reagents and antibodies

The immunology inflammation screening library (L4100), dorsomorphin (S7840) and 3-methyladenine (3-MA, S2767) were purchased from Selleck (Shanghai, China). Anti-IPMK (ab169241) antibody were purchased from Abcam (Shanghai, China). Anti-LC3B (3868T), Atg7 (8558T), AMPK (5831T), and p-AMPK (2535T) antibodies were purchased from Cell Signaling Technology (CST, Shanghai, China). Anti-β-actin (HRP-60008), CDK2 (66950-1-Ig), CyclinA2 (18202-1-AP), ULK1 (20986-1-AP), Beclin1 (66665-1-Ig), Atg16 (19812-1-AP), Caspase-9 (10380-1-AP), and Caspase 8 (66093-1-Ig) antibodies were purchased from Proteintech (Wuhan, Hubei, China).

### Cell culture

Human osteosarcoma cell lines (MNNG, MG63, and U2OS) were obtained from the Cell Bank of the Chinese Academy of Sciences (Shanghai, China). Cells were cultured in Dulbecco's modified Eagle's medium (DMEM) or RPMI-1640 medium supplemented with 10% fetal bovine serum (Gibco, Grand Island, NY, USA), 1% penicillin-streptomycin and 1% nonessential amino acids (NEAAs) under standard culture conditions (37°C, 95% humidified air and 5% CO_2_). The medium was changed every 2 days.

### High-throughput screening of candidate anti-inflammatory agents

The inhibition rate of every compound was assayed at a concentration of 50 µmol/L in three osteosarcoma cell lines at 48 h. The control group was treated with an equal amount of DMSO (dimethyl sulfoxide). A CCK-8 assay was used to determine cell viability.

### Cell viability assay

Osteosarcoma cells were incubated in 96-well plates for 24 h at a density of 5×10^3^ cells/well in 200 µL culture medium. Then, the cells were treated with different doses of ebastine (the range was from 0 to 30 µM, with 4 µM intervals). After 48 h, cell viability was detected using the Cell Counting Kit-8 assay (CCK-8; Dojindo, Kumamoto, Japan). Ten microliters of CCK8 working solution was added to each well for approximately 2 h, and the absorbance value of each well was measured at 450 nm using an enzyme-labeled instrument. Then, GraphPad Prism 8 software was used to calculate the half-maximal inhibitory concentration (IC50) of ebastine.

### CCK-8 assay

Osteosarcoma cells were seeded into 6-well plates (3×10^5^ cells/well) and treated with the IC30 or IC50 of ebastine for 48 h. The cells were digested and seeded into 96-well plates (5000 cells/well), treated with culture medium supplemented with 10% FBS and evaluated by CCK-8 assay for 5 days. The control group was treated with an equal amount of DMSO. The Cell Counting Kit-8 assay is a rapid, highly sensitive, non-radioactive colorimetric detection kit based on WST-8 that is widely used in cell proliferation and cytotoxicity. The CCK-8 solution can be added directly to the cell sample without pre-preparation of various components. In the presence of electron coupling reagent, WST-8 can be reduced to orange-yellow by some dehydrogenases in mitochondria. The more and faster the cell proliferates, the darker the color. For the same cells, the depth of the color and the number of cells are linear relationship.

### Clone formation assay

After treatment with ebastine at the IC30 or IC50 for 48 h, cells were cultured in 6-well plates at a density of 1000 cells/well and incubated under standard culture conditions for 10 days. The control group was treated with an equal amount of DMSO. Next, the medium was removed, and the cell clones were washed with PBS (phosphate-buffered saline). Then, they were fixed in 4% paraformaldehyde and dyed with 0.1% crystal violet. Finally, cell colonies were quantified.

### Cell migration and invasion assays

Cell migration and invasion ability were assessed through Transwell filter chambers (Corning, New York, NY, USA), which were 8-mm pore size chamber inserts in a 24-well plate. For the migration assay, 48 h after the IC30 or IC50 ebastine treatment, 200 μL of serum-free medium containing 5×10^4^ MNNG and U2OS cells or 1×10^5^ MG63 cells was dropped into the upper chambers. For the invasion assay, 1×10^5^ MNNG, U2OS cells or 2×10^5^ MG63 cells were placed into the upper chambers, which were coated with Matrigel diluted in serum-free culture medium. Separately, 800 μL of culture medium supplemented with 10% FBS (fetal bovine serum) was added to the lower chambers. The control group was treated with an equal amount of DMSO. After incubation at 37°C, the cells on the bottom surface of the membrane were stained and counted.

### Wound-healing assay

Osteosarcoma cells were seeded into 6-well plates to reach 90% confluence. Then, the cells were scraped/wounded using a yellow tip, treated with different drugs (the IC30 or IC50 of ebastine) and measured at 0 h, 12 h, and 24 h by microscopy. The control group was treated with an equal amount of DMSO.

### Flow cytometry analysis of apoptosis and cell cycle distribution

Cells were seeded into 6-well plates (3×10^5^ cells/well) and then exposed to the IC30 or IC50 of ebastine, 3-MA, and dorsomorphin or their combinations. After 48 h, cells were collected, washed, and stained according to the manufacturer's guidelines (Annexin V-FITC Apoptosis Detection Kit; Beyotime, China). The control group was treated with an equal amount of DMSO. Then, samples were read on a flow cytometer (BD Biosciences, Franklin Lakes, NJ, USA). For the cell cycle assay, cells were treated as mentioned above, collected according to the manufacturer's guidelines (Cell Cycle and Apoptosis Analysis Kit; Beyotime, China) and analyzed by flow cytometry.

### Western blot analysis

The tumor tissue was weighed and added to RIPA lysis buffer (radioimmunoprecipitation assay lysis buffer, Thermo Fisher, Waltham, USA) at a ratio of 1:8 (1 g tissue plus 8 ml RIPA lysis buffer). Protease and phosphatase inhibitors were added at a ratio of 1:100. The tissue was homogenized with a homogenizer on ice, mixed and placed into an EP (Eppendorf) tube. In addition, cells were scraped into EP tubes using a cell scraping tools and mixed with RIPA lysis buffer. All procedures were performed on ice. After measuring the protein concentration using BCA (bicinchoninic acid assay) protein assays (Thermo Fisher, Waltham, USA), 5× SDS loading buffer was added to each EP tube and heated at 95°C for 5 min. Proteins were separated by 8%-12% SDS-PAGE (sodium dodecyl sulfate polyacrylamide gel electrophoresis) and transferred to polyvinylidene fluoride (PVDF) membranes (Millipore, Massachusetts, USA). The protein mass marker was a mixture of proteins from 10 kDa to 245 kDa (Yeasen, Shanghai, China, 20352ES76). Membranes were incubated with 1:1000-5000 primary antibodies at 4°C overnight, followed by incubation with secondary antibodies at room temperature for 1 h. Finally, the ECL (electrochemiluminescence) detection system (SmartChemi 420, Beijing, China) was used to measure the immune reaction zone. Western blot images were quantified using ImageJ software.

### RNA extraction and real-time polymerase chain reaction (RT-PCR) assay

Total RNA was extracted using TRIzol reagent (Invitrogen, California, USA) according to the manufacturer's protocol. First-strand cDNA was synthesized using a PrimeScript RT Reagent Kit (Takara, Dalian, China). The cDNA templates were combined with SYBR Green premix with Rox II (Takara, Dalian, China) to perform quantitative real-time polymerase chain reaction (qRT-PCR). Information on the primer sequences used is presented in Supplementary Table 1.

### Animal experiments

Female BALB/c nude mice (20 g/mouse) were used in animal studies, and all animals were maintained in specific pathogen-free (SPF) conditions. MNNG cells were subcutaneously injected into the left scapula of nude mice (1×10^6^ cells/mouse). The mice were then randomized into the following 3 groups: (1) 0.9% NaCl (0.9% NaCl in 200 μL, po/day/mouse); (2) ebastine (ebastine 0.5 mg/day); and (3) ebastine (ebastine 1 mg/day). For lung metastasis studies, the mice were injected with 2×10^6^ MNNG cells into the tail vein. After 1 week, the mice were randomized into 2 groups: (1) the 0.9% NaCl (0.9% NaCl in 200 μL, po/day/mouse) and (2) ebastine (ebastine 1 mg/day). The body weights and tumor volumes of the mice were measured every 3 days. After 30/45 days, the mice were euthanized, and the xenograft tumors from each animal were weighed and analyzed. The lung tissues of each animal were weighed and fixed in 10% formalin for histological analysis. The fixed samples were embedded in paraffin and made into tissue sections. Some sections were stained with hematoxylin and eosin (HE), and others were stained with immunohistochemistry (IHC). All animal experiments were performed according to the guidelines approved by the Shanghai Medical Experimental Animal Care Commission.

### mRFP-GFP-LC3-expressing cell generation and fluorescent LC3 puncta analysis

To perform image-based analysis for autophagy, cells were infected with tandem GFP-RFP-LC3 adenovirus for 24 h according to the manufacturer's instructions. Then, cells were treated and imaged for green fluorescent protein (GFP) and red fluorescent protein (RFP) using a Fluoview FV1000 microscope (Olympus, Tokyo, Japan).

### Transmission electronic microscopy

Cells treated with or without ebastine were harvested, fixed, dehydrated, embedded in Epon, stained and observed. Images were acquired using an HT7700 transmission electronic microscope (HITACHI, Tokyo, Japan) to observe autophagic vacuoles. The number of autophagic vacuoles (AVs) in each cell was quantified in 20 randomly selected cells in each group.

### Cell transfection and efficiency test

Lipofectamine 2000 (Invitrogen, California, USA) was used for plasmid transfection. SiRNAs were designed and synthesized by RiboBio (Guangzhou, China). Cells were plated at 60-70% confluence in 6-well plates and transfected with siRNA using Lipofectamine 2000 Reagent according to the manufacturer's protocol. The transfection efficiency was determined by qRT-PCR and western blot.

### IHC analysis

A standard IHC staining procedure was implemented. Briefly, paraffin-embedded sections were cut at 4 μm, dewaxed in xylene, and heated in a microwave at 60 °C for 20 min in EDTA buffer (pH 9.0) for antigen retrieval. For each slide, endogenous peroxidase activity was blocked by a 10 min incubation in 0.3% H_2_O_2_ followed by incubation at 37 °C with a 1:100 dilution of the primary antibody KSR1. Slides were rinsed three times in PBS, incubated for 30 min with an EnVision staining kit (DAKO), followed by three additional washes in PBS, and color was developed over 3-10 min in a moist chamber at room temperature using 3,3′-diaminobenzidine. Slides were counterstained in hematoxylin and dehydrated in a graded ethyl alcohol series (70%, 90%, and 100%). Assessment of IHC staining was independently performed by two expert pathologists. Any discordance was resolved through discussion and consensus.

### Statistical analysis

All data are representative at least three independent experiments. Data are expressed as mean ± standard deviation (SD). Statistical analysis was conducted using GraphPad Prism 8 software (San Diego, CA, USA). Differences between experimental groups and control groups were calculated by Student's t-test, and *P*<0.05 was considered statistically significant (*P<0.05; **P<0.01; ***P<0.001).

## Results

### High-throughput screening generates candidate antitumor agents for osteosarcoma therapy

**Figure 1:**
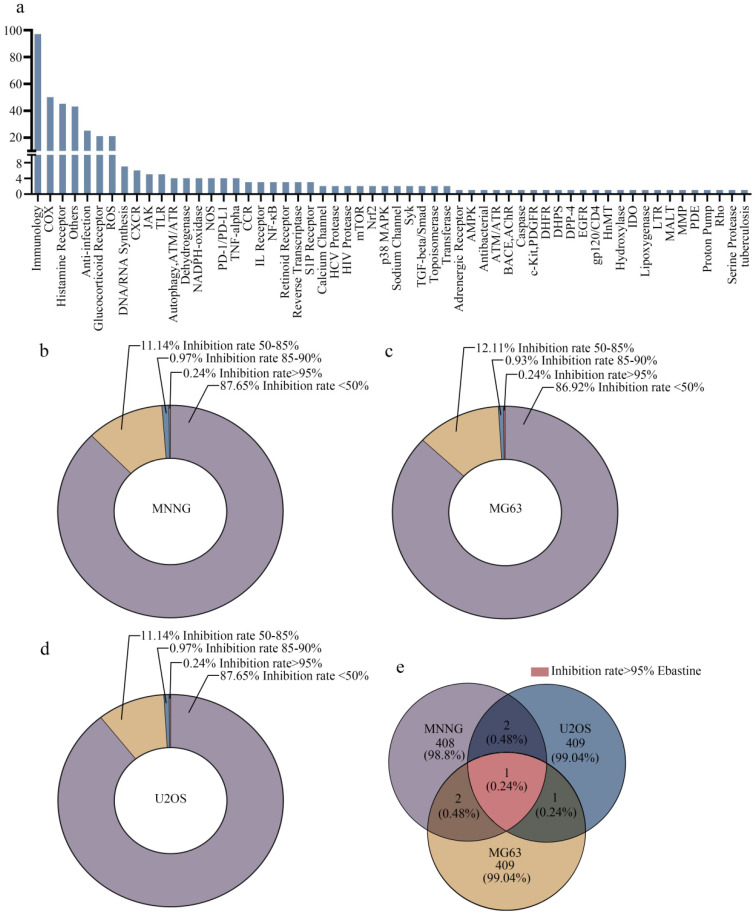
** High-throughput screening generates candidate anti-inflammatory agents for osteosarcoma therapy.** (**a**) The composition of this immunology inflammation screening library (L4100). (**b-d**) The inhibition rate of every compound in all three osteosarcoma cell lines. (**e**) The inhibition rate > 95% in three osteosarcoma cell lines was achieved by ebastine.

In this study, 413 compounds from libraries consisting of approved drugs, drug candidates in clinical trials and pharmacologically active compounds were screened to identify new inhibitors against osteosarcoma. The compounds focused on immunology and inflammation. The composition of this immunology/inflammation compound library primarily included targets such as immunology, COX, histamine receptor, anti-infection, glucocorticoid receptor, ROS, DNA/RNA synthesis, CXCR, and PD-1/PD-L1 (Fig. 1a). Then, the inhibition rate of every compound was assayed at a concentration of 50 µmol/L in three osteosarcoma cell lines. In MNNG cells, 87.65% of library compounds showed < 50% inhibition, 11.14% of compounds showed 50%-85% inhibition, 0.97% of compounds showed 85-90% inhibition, and 0.24% of compounds showed > 95% inhibition (Fig. 1b). In MG63 cells, 86.92% of library compounds showed < 50% inhibition, 12.11% of compounds showed 50%-85% inhibition, 0.93% of compounds showed 85-90% inhibition, and 0.24% of compounds showed > 95% inhibition (Fig. 1c). In U2OS cells, 87.65% of library compounds showed < 50% inhibition, 11.14% of compounds showed 50%-85% inhibition, 0.97% of compounds showed 85-90% inhibition, and 0.24% of compounds showed > 95% inhibition (Fig. 1d). Thus, the results showed that the inhibition rate of most compounds was less than 85%. The inhibition rate of only 3-4 compounds reached 85%-90%. Finally, only one compound (ebastine, an H1-histamine receptor antagonist) had an inhibition rate of 95% in all three osteosarcoma cell lines (Fig. 1e; S1a-c).

### Ebastine exhibits antitumor activity in osteosarcoma cells in a concentration-dependent manner* in vitro*

**Figure 2:**
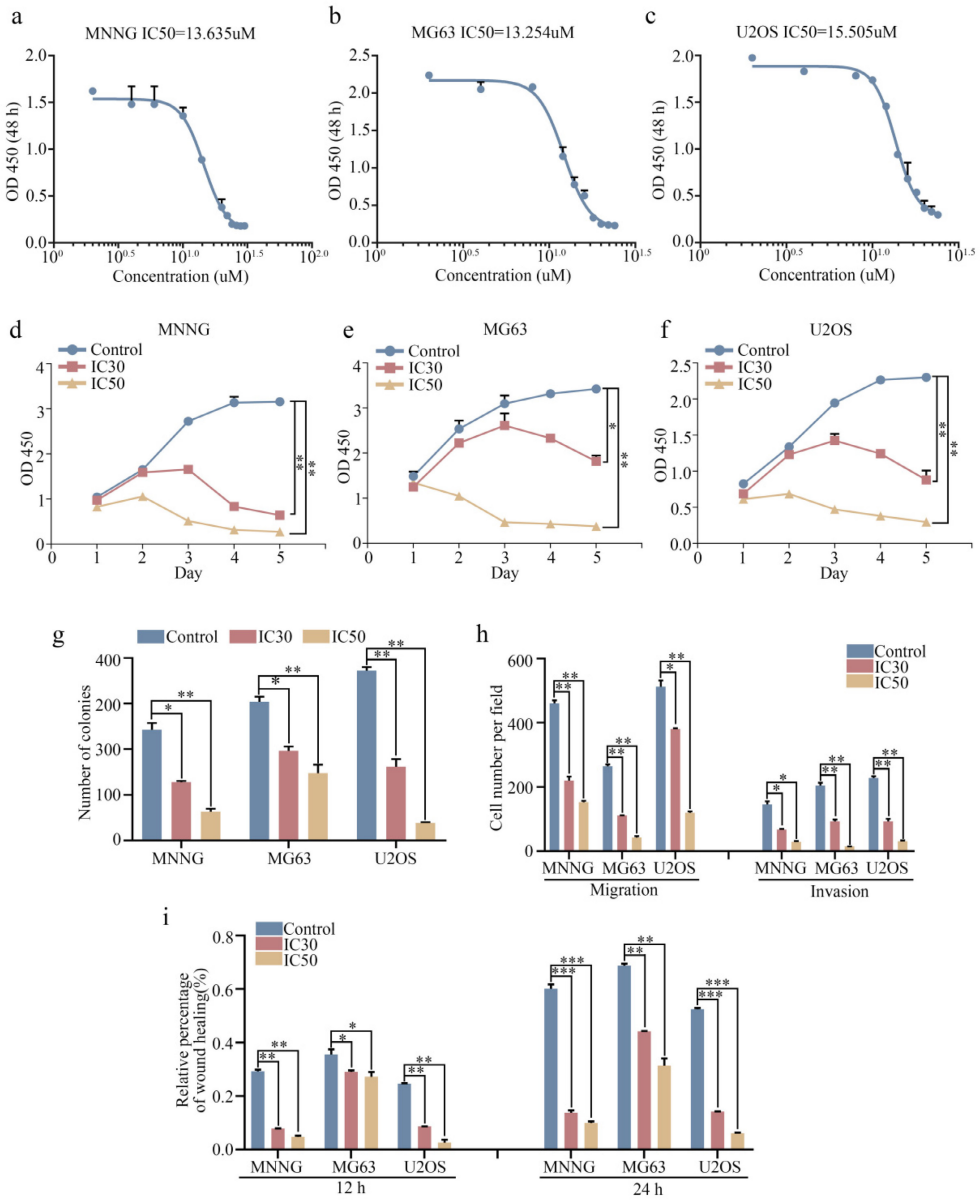
** Ebastine exhibits antitumor activity in osteosarcoma cells *in vitro.*** (**a-c**) *In vitro*, osteosarcoma cells were treated with ebastine at different concentrations (the range was from 0 to 30 µM) for 48 h, and the IC50 was calculated. (**d-f**) A CCK-8 assay was used to detect the proliferation of osteosarcoma cells after treatment with the IC30 and IC50 of ebastine. (**g**) Colony formation assays in osteosarcoma cells after treatment with the IC30 and IC50 of ebastine. (**h**) Transwell migration and invasion assays in osteosarcoma cells after treatment with the IC30 and IC50 of ebastine. (**i**) A wound-healing assay was used to determine cell migration after treatment with the IC30 and IC50 of ebastine. Data are shown as the means ± SD from at least three independent experiments. Statistical analysis was performed using Student's t-test. Error bars represent the SEM. ** P<0.01; *** *P*<0.001.

We next investigated the antitumor effect of ebastine against osteosarcoma cells (MNNG, MG63, and U2OS) *in vitro*. The 30% inhibitory concentration (IC30) and 50% inhibiting concentration (IC50) of ebastine at 48 h in the three cell lines were analyzed using GraphPad Prism8. The IC50 values for MNNG, MG63, and U2OS cells were 13.635 µM, 13.254 µM and 15.505 µM, respectively (Fig. 2a-c). The IC30 values for MNNG, MG63, and U2OS cells were 9.956 µM, 10.221 µM and 11.592 µM, respectively. Next, we used the IC30 and IC50 of ebastine to evaluate the antiproliferative capacity of osteosarcoma cells. The results showed that ebastine significantly inhibited the growth of osteosarcoma cells (Fig. 2d-f). And the IC30 and IC50 of ebastine can not affect the growth of osteoblast cell hFOB 1.19 (Fig. S1d). Similarly, findings from the colony formation assay revealed that ebastine attenuated the formation of cell colonies (Fig. 2g; S2a). Furthermore, Transwell assays and wound-healing assays were conducted to determine whether ebastine inhibited the migration and invasion of osteosarcoma cells. Ebastine significantly suppressed the migration and invasion of osteosarcoma cells (Fig. 2h; S2b), and the wound gap was wider in the ebastine treatment group than in the control group (Fig. 2i; S2c-e). Thus, these results demonstrated that ebastine exhibited a suppressive effect in osteosarcoma cells in a concentration-dependent manner *in vitro*.

### Ebastine affects the osteosarcoma cell cycle and apoptosis *in vitro* and inhibits osteosarcoma cell growth and metastasis *in vivo*

**Figure 3:**
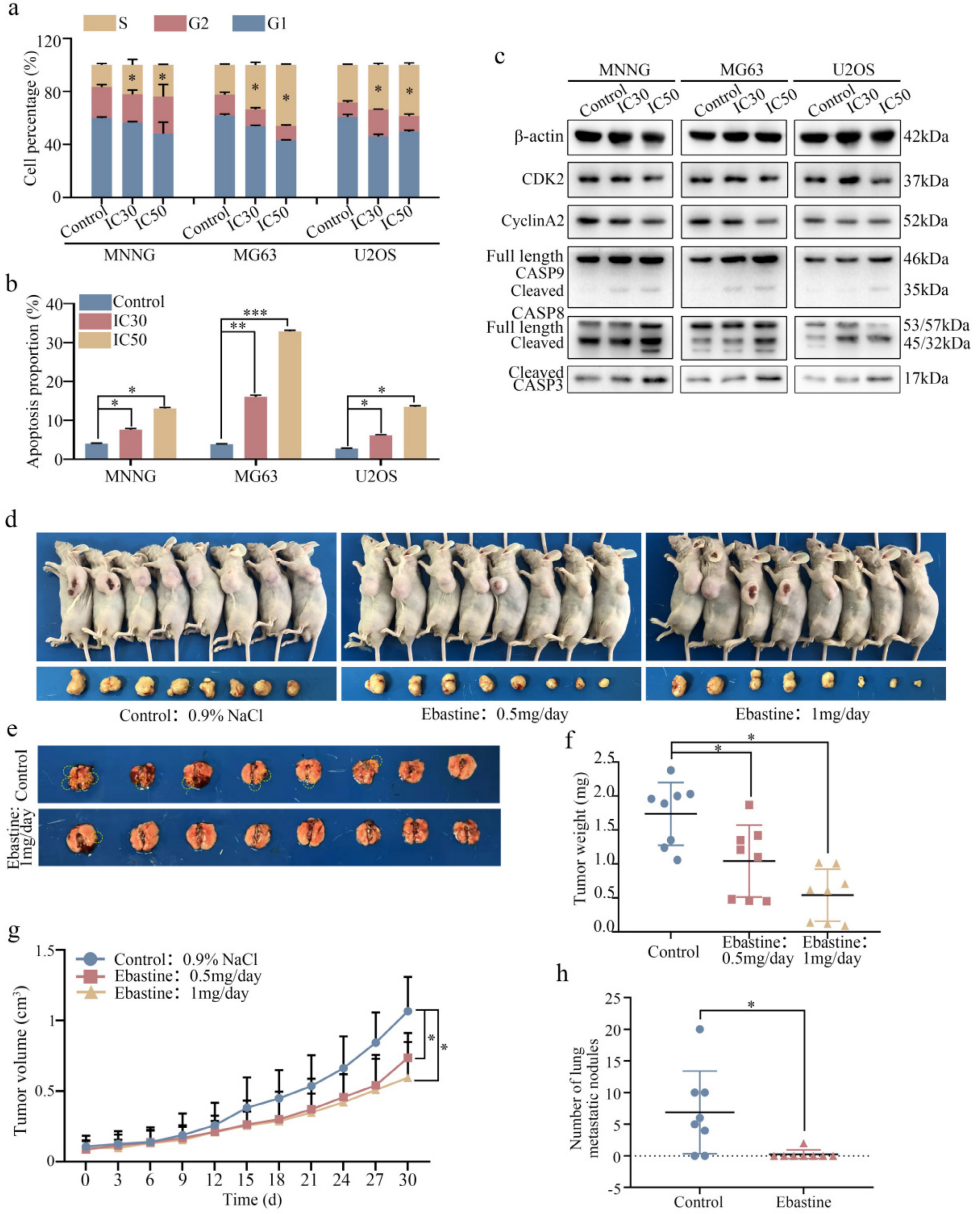
** Ebastine affects the osteosarcoma cell cycle and apoptosis *in vitro* and inhibits osteosarcoma cell growth and metastasis *in vivo.*** (a-b) Flow cytometry histograms of the cell cycle and apoptosis change in response to treatment with the IC30 and IC50 of ebastine for 48 h. (c) After treatment with ebastine, representative results of CDK2, CyclinA2, full length and cleavage of caspase‐9, ‐8, -3 protein levels were determined by western blot analysis. (d) Image of tumor-bearing mice after treatment with ebastine. (e) Lung metastases formed by MNNG cells in the ebastine treatment and control groups. (f) Diagram showing tumor weights in the subcutaneous tumor model. (g) Growth curve drawn by measuring tumor volumes. (h) Statistical analysis of lung metastasis nodules in the lung metastasis model. Data are shown as the means ± SD from at least three independent experiments. Statistical analysis was performed using Student's t-test. Error bars represent the SEM. *P<0.05; ** *P*<0.01; *** *P*<0.001.

Next, we examined the cell cycle distribution and apoptosis initiation changes in response to ebastine treatment. Flow cytometry was used to evaluate cell cycle distribution. As shown in Figure 3a, osteosarcoma cells were arrested at S phase after treatment with IC30 and IC50 ebastine for 48 h (Fig. S3a). Annexin V‐FITC/PI double‐staining analysis showed that the percentages of apoptotic cells were dramatically increased after incubation with IC30 and IC50 ebastine for 48 h (Fig. 3b; S3b). Western blot analysis demonstrated that expression of CDK2 and Cyclin A2, which are the primary cell cycle regulators in S phase, was significantly downregulated after treatment with IC30 and IC50 of ebastine (Fig. 3c; S3c). Meanwhile, expression of cleaved caspase-8, caspase-9, and caspase-3 was increased in response to exposure to ebastine (Fig. 3c; S3c). Then, we established a subcutaneous transplantation model and a lung metastasis model by transplanting MNNG cells into nude mice subcutaneously or injecting them into the lateral tail veins of nude mice to confirm the potential antitumor effect of ebastine *in vivo*. The results demonstrated that ebastine inhibited tumor growth and lung metastasis (Fig. 3d, e). Ebastine significantly decreased both tumor weight (Fig. 3f) and tumor volume (Fig. 3g) and had no effect on the body weight of the mice (Fig. S4a) in the subcutaneous transplantation model. Ebastine significantly decreased the number of lung metastatic nodules (Fig. 3h, S4c) and had no effect on body weight of mice in the lung metastasis model (Fig. S4b). In addition, the lung weight of the ebastine group was higher than that of the control group (Fig. S3d). HE staining of the heart, liver, spleen, lung and kidney showed that there was no toxicity in ebastine-treated mice (Fig. S4e). Taken together, we concluded that ebastine dramatically hindered tumorigenesis and lung metastasis of osteosarcoma cells *in vitro* and *in vivo*.

### Ebastine promotes autophagy in osteosarcoma cells

**Figure 4:**
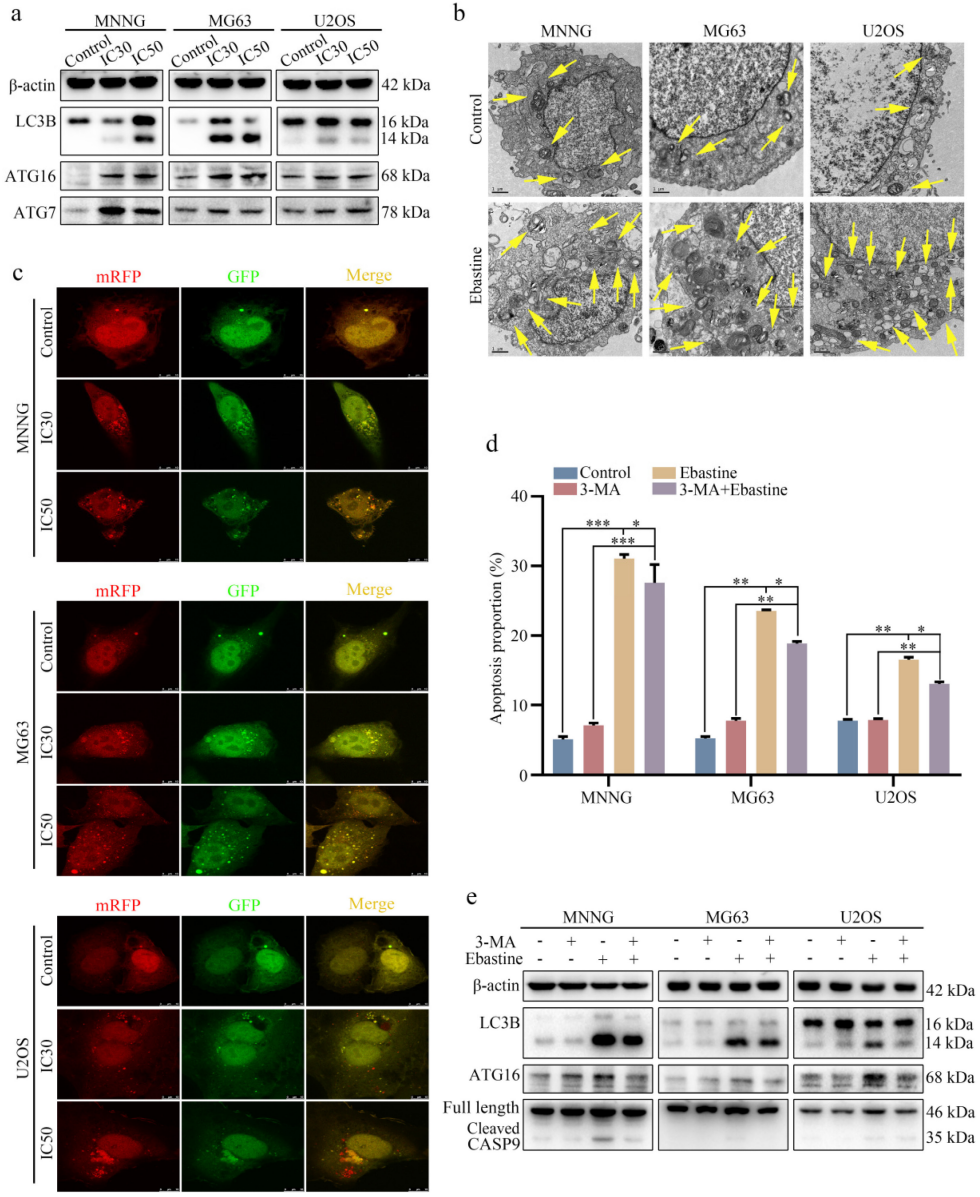
** Ebastine promotes autophagy in osteosarcoma cells.** (a) After treatment with ebastine for 48 h, the protein levels of LC3B, ATG16 and ATG7 were assayed by western blot. (**b**) Autophagosome-like structures (indicated by the yellow arrows) were assayed by TEM. (**c**) LC3 puncta were analyzed using the mRFP-GFP-LC3 construct. (**d**) Flow cytometry was used to analyze apoptosis after treatment with ebastine for 48 h with or without 3-MA (0.5 mM, 2 h). (**e**) After treatment with ebastine for 48 h with or without 3-MA, autophagy activity indicated by LC3 levels was analyzed by western blot. Data are shown as the means ± SD from at least three independent experiments. Statistical analysis was performed using Student's t-test. Error bars represent the SEM. *P<0.05; ** P<0.01; *** *P*<0.001.

To further elucidate the biological function of ebastine in osteosarcoma, we examined the autophagy-inducing activity of ebastine in osteosarcoma cell lines. Autophagy-related proteins were analyzed using western blotting, and the results revealed that ebastine caused a significant increase in LC3B, ATG7 and ATG16 (Fig. 4a; S6a). In addition, to directly demonstrate autophagosome formation, we used TEM to observe numerous large autophagic vacuoles in the cytoplasm, in which the vacuolar contents were degraded, providing evidence for the impact of ebastine in the regulation of autophagic formation in osteosarcoma after treatment with the IC50 of ebastine (Fig. 4b). Next, we utilized the mRFP-GFP-LC3 adenovirus construct to further confirm autophagy induction by puncta formation. In the present study, after infection with the mRFP-GFP-LC3 adenovirus, we observed the successful introduction of this adenovirus, showing both fluorescent proteins (Fig. 4c). The numbers of yellow and free red puncta were both significantly higher after treatment with ebastine, indicating increased autophagosomes and autolysosomes (Fig. S5a). In addition, the autophagy inhibitor 3-MA was applied to osteosarcoma cells, and ebastine-induced apoptosis was blocked by 3-MA (Fig. 4d; S5b). As shown in Fig. 4e, 3-MA diminished LC3B, ATG16, and cleavage of caspase‐9 induced by ebastine (Fig. 4e; S6b). Therefore, these data suggested that ebastine promoted autophagy in osteosarcoma cells.

### Ebastine promotes autophagy and induces apoptosis through activation of the AMPK/ULK1 signaling pathway

**Figure 5:**
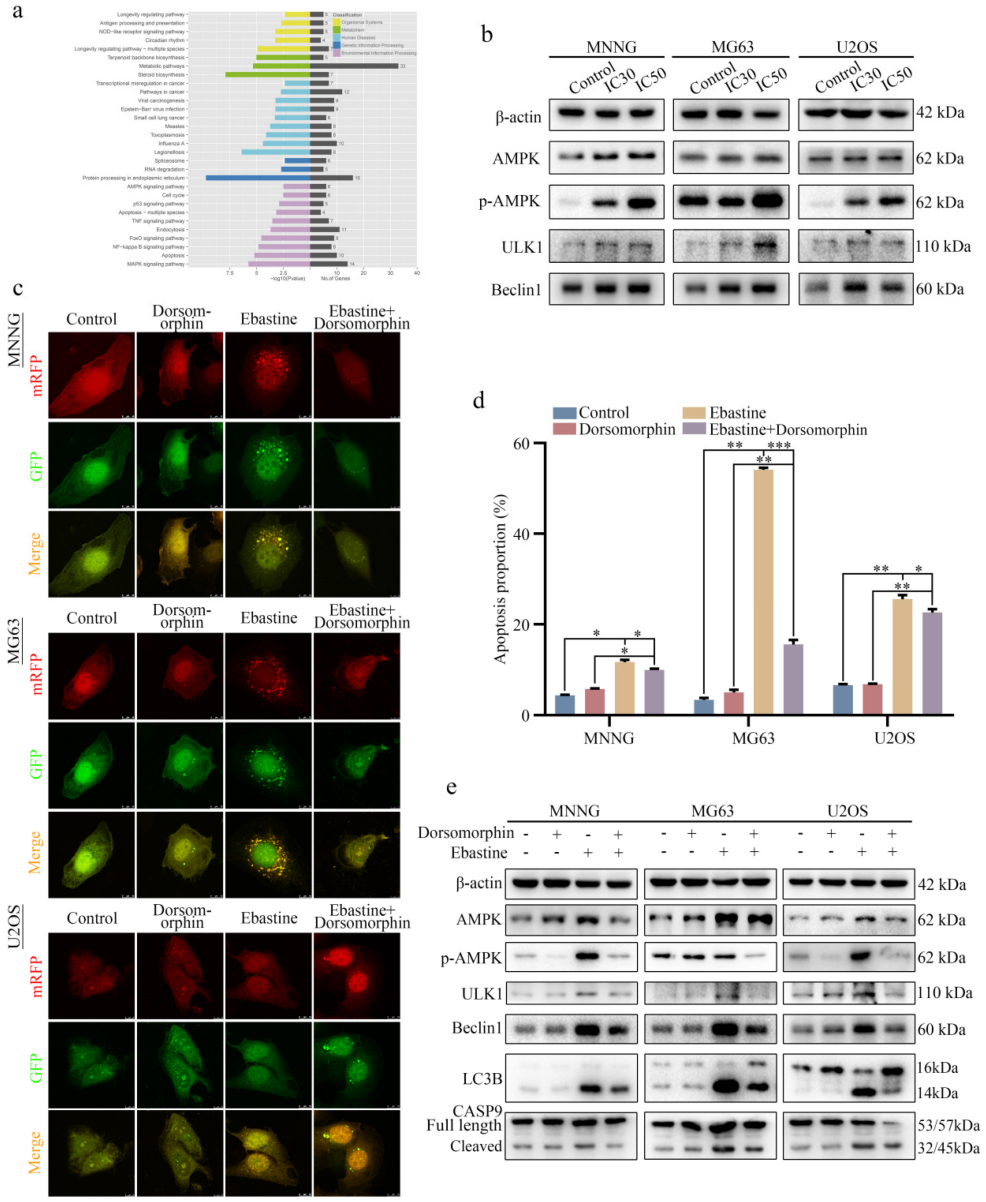
** Ebastine promotes autophagy and induces apoptosis through activation of the AMPK/ULK1 signaling pathway.** (**a**) KEGG pathway analysis of the differentially expressed genes in ebastine-treated MNNG cells and control cells. (**b**) Representative blots showing the protein expression of AMPK, p-AMPK, ULK1 and Beclin1 after treatment with ebastine at the IC30 and IC50 values. (**c**) LC3 puncta were analyzed using the mRFP-GFP-LC3 construct after treatment with ebastine for 48 h with or without dorsomorphin in osteosarcoma cells. (**d**) Apoptosis was assessed by flow cytometry analysis after treatment with ebastine for 48 h with or without dorsomorphin in osteosarcoma cells. (**e**) western blot analysis showing the protein expression of AMPK, p-AMPK, ULK1, Beclin1, LC3B and cleavage of caspase‐9 in osteosarcoma cells after treatment with ebastine for 48 h with or without dorsomorphin in osteosarcoma cells. Data are shown as the means ± SD from at least three independent experiments. Statistical analysis was performed using Student's t-test. Error bars represent the SEM. *P<0.05; ** *P*<0.01; *** *P*<0.001.

To explore the underlying signaling pathway affected by ebastine, RNA-seq analysis was performed in MNNG cells after treatment with ebastine. The volcano plot analysis revealed 519 upregulated and 460 downregulated genes that were identified and quantified (Fig. S7a). Then, 15 upregulated genes and 11 downregulated genes were chosen to verify the results of the RNA-seq analysis, and the changes are shown in the heat map (Fig. S7b, c). Next, KEGG pathway analysis was performed based on the RNA-Seq data. The results showed that ebastine was related to the AMPK signaling pathway, which plays a pivotal role in autophagy (Fig. 5a). Next, we examined the changes of key node proteins of this pathway and autophagy-related proteins by western blot. The results showed that the AMPK, p-AMPK, ULK1 and Beclin1 proteins were significantly upregulated (Fig. 5b; S7d). Moreover, we used the AMPK inhibitor dorsomorphin to determine the role of AMPK/ULK1 signaling in ebastine-mediated antitumor activity. When dorsomorphin was combined with ebastine, autophagic flux analysis showed that the numbers of yellow and free red puncta were both decreased (Fig. 5c; S7e), and flow cytometry showed that apoptosis was reduced (Fig. 5d; S8a). In contrast, expression of AMPK, p-AMPK, ULK1, Beclin1, LC3B and cleavage of caspase‐9 were significantly restored (Fig. 5e; S8b). These results indicated that dorsomorphin treatment restored ebastine-induced cell autophagy and apoptosis. Thus, ebastine promoted autophagy and induced apoptosis through activation of the AMPK/ULK1 pathway.

### Knockdown of IPMK restores the induction of autophagy and activation of the AMPK/ULK1 signaling pathway in response to ebastine treatment

**Figure 6:**
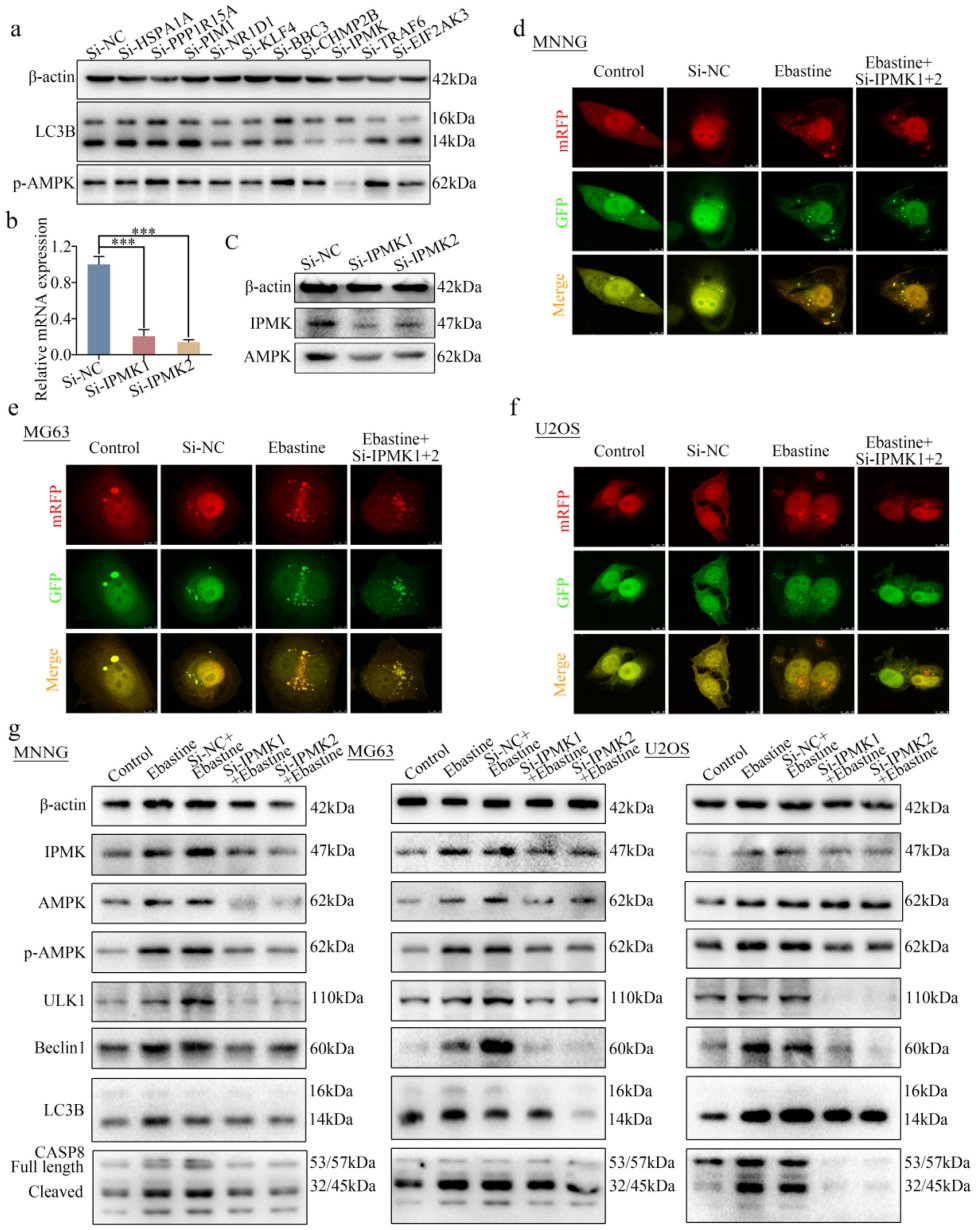
** Knockdown of IPMK restores the induction of autophagy and activation of the AMPK/ULK1 signaling pathway by ebastine treatment.** (**a**) Expression of LC3B and p-AMPK was analyzed by western blot after knockdown of 10 genes in MNNG cells. (**b**) After knockdown of IPMK in MNNG cells, the mRNA expression of IPMK was determined by qRT-PCR. (**c**) Protein expression of IPMK and AMPK was analyzed by western blot after knockdown of IPMK in MNNG cells. (**d-f**) Osteosarcoma cells were treated with ebastine for 48 h with or without knockdown of IPMK, and LC3 puncta were analyzed by the mRFP-GFP-LC3 construct. (**g**) Protein levels of apoptosis, autophagy, IPMK, AMPK and p-AMPKK in osteosarcoma cells treated with ebastine for 48 h with or without knockdown of IPMK. Data are shown as the means ± SD from at least three independent experiments. Statistical analysis was performed using Student's t-test. Error bars represent the SEM. *P<0.05.

To further determine how ebastine regulates the AMPK/ULK1 pathway in osteosarcoma, 21 differentially expressed genes in the RNA-seq data were found to be related to autophagy in PubMed. First, we examined expression of these 21 genes in response to treatment with ebastine in three osteosarcoma cell lines. Ten genes were found to be simultaneously upregulated in all three osteosarcoma cell lines (Fig. S9a-c). Next, we knocked down the 10 genes via independent siRNAs in MNNG cells (Fig. S9d). Then, we tested the changes in LC3B and p-AMPK using western blotting. The results showed that knockdown of IPMK significantly decreased expression of LC3B and p-AMPK (Fig. 6a). A previous study reported that IPMK interacted with AMPK 44. Then, we designed two independent siRNAs to knock down IPMK and examined the expression of AMPK, which was found to be downregulated after knockdown of IPMK (Fig. 6b, c). Finally, to test the role of IPMK in ebastine-mediated antitumor activity, we examined the activity of autophagy in si-IPMK-transfected cells after ebastine treatment. After infection with the mRFP-GFP-LC3 adenovirus, the numbers of yellow and free red puncta in the si-IPMK+ebastine group were both significantly lower than those in the ebastine group, indicating that autophagosomes and autolysosomes had decreased in the three osteosarcoma cell lines (Fig. 6d-f; S9e). In addition, western blot analysis revealed that IPMK knockdown restored the upregulation of AMPK, p-AMPK, ULK1, Beclin1, LC3B and cleavage of caspase‐8 caused by ebastine treatment (Fig. 6g; S10a). These results revealed that IPMK knockdown restores the induction of autophagy and activation of the AMPK/ULK1 signaling pathway in response to ebastine treatment.

### Ebastine exerts antitumor activity through the IPMK/AMPK/ULK1 signaling pathway *in vivo*

**Figure 7:**
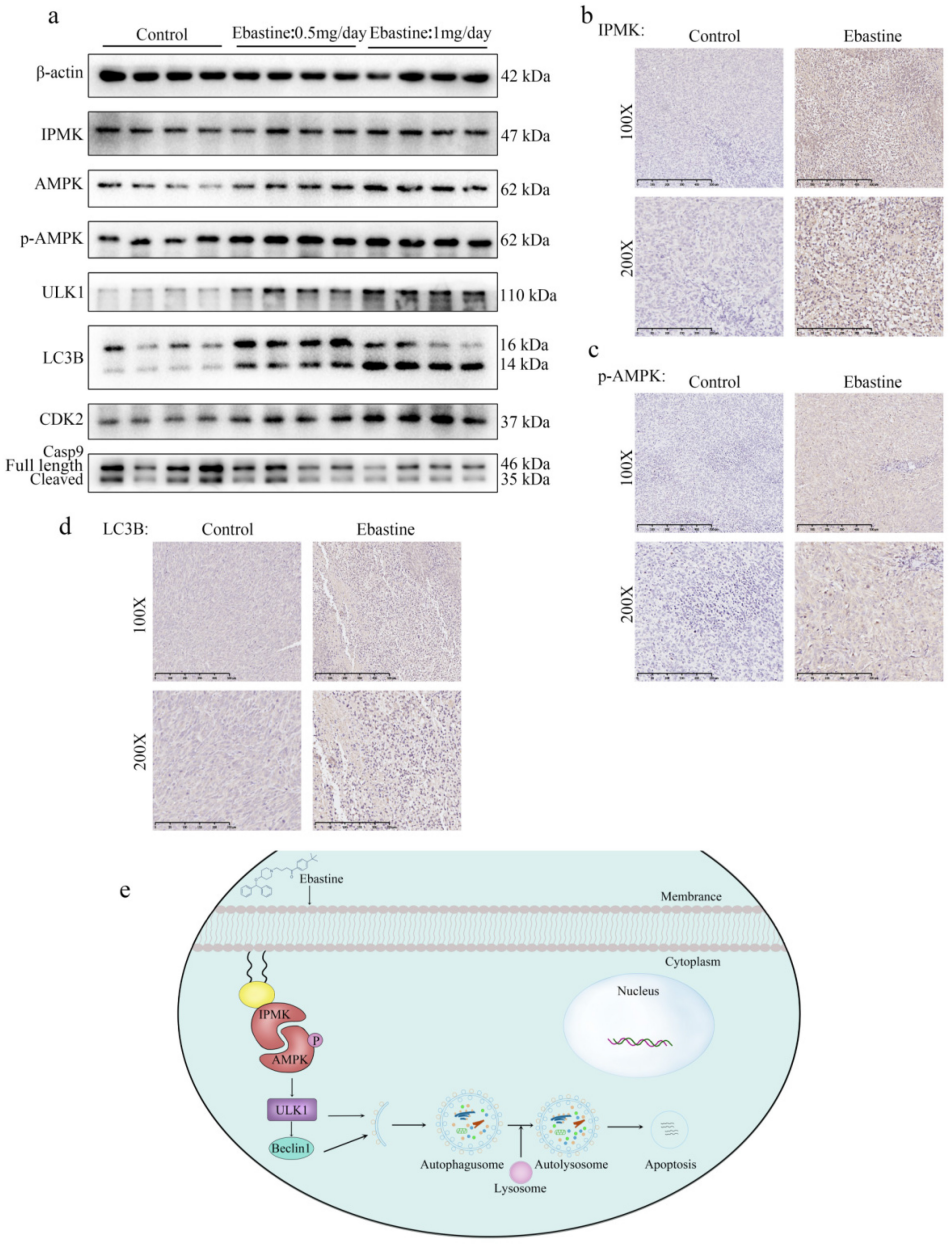
** Ebastine exerts antitumor activity through the IPMK/AMPK/ULK1 signaling pathway *in vivo*.** (**a**) Protein expression of IPMK, AMPK, p-AMPK, ULK1, LC3B, and CDK2 and cleavage of caspase‐9 were analyzed by western blot in tumor tissues*.* (**b-d**) IHC staining of related proteins. (**e**) The diagram of the mechanism of ebastine anti-osteosarcoma. Statistical analysis was performed using Student's t-test. Error bars represent the SEM. *P<0.05, ** P<0.01; *** *P*<0.001.

Finally, to further determine the role of IPMK and the AMPK/ULK1 pathway in ebastine-mediated antitumor activity* in vivo*, we examined expression of IPMK and the AMPK/ULK1 pathway in subcutaneous tumor tissues. After mice were euthanized, the three groups' specimens were fixed in formalin for IHC staining, and four samples of each group were submerged in liquid nitrogen to achieve cryo-etching for western blot analysis. The western blot results showed that ebastine upregulated expression of IPMK, AMPK, p-AMPK, ULK1, LC3B compared to the control group (Fig. 7a; S10b). Similarly, the IHC analysis indicated that the staining of IPMK, p-AMPK, ULK1, LC3B and caspase‐9 was higher, and the staining of CDK2 was lower in the ebastine group than in the control group (Fig. 7b-d; S11a-c). However, the expression cleavage of caspase‐9 and CDK2 in tissue were not consistent with in cellular. This phenomenon might be related to differences between tissue proteins and cellular proteins. Taken together, we concluded that ebastine exerts antitumor activity through the IPMK/AMPK/ULK1 signaling pathway* in vivo.* And the diagram of the mechanism of ebastine anti-osteosarcoma was shown in Figure 7e.

## Discussion

Osteosarcoma is the most common primary malignant tumor of bone and is prone to local invasion and metastasis 1. Although the combination of surgery and chemotherapy has greatly improved the prognosis of patients with osteosarcoma, the 5-year survival rate has not made significant progress in recent decades 10. It has been demonstrated that immunotherapy is a promising therapeutic strategy for human malignant tumors that improves the understanding of the immune response to osteosarcoma 26. Inflammation is of great importance in tumorigenesis, malignant conversion, and antitumor immunity in the human body. Therefore, immunotherapy is an increasingly attractive option for treating osteosarcoma patients 27-28. In addition to immune regulation, anti-inflammatory agents also directly inhibit the proliferation of osteosarcoma cells. A case report describes an osteosarcoma patient receiving the cyclooxygenase 2 (COX2) inhibitors celelecoxib and thalidomide one month after treatment with lung metastases. It has also been found that celecoxib and thalidomide inhibit proliferation of osteosarcoma cells in a dose-dependent manner 29. These studies support anti-inflammatory agents as potential therapeutic strategies for osteosarcoma patients.

Here, we focused on the direct antitumor activity of a panel of bioactive compounds in osteosarcoma. The composition of this immunology/inflammation compound library primarily included targets such as immunology, COX, histamine receptor, anti-infection, glucocorticoid receptor, and ROS. After screening osteosarcoma cells, it was found that the inhibition rate of most compounds was less than 85%. Only 3-4 compounds exhibited an inhibition rate that reached 85%-90%. In addition, only one compound (ebastine, an H1-histamine receptor antagonist) had an inhibition rate of 95% in all three osteosarcoma cell lines. Ebastine, acting predominantly via the H1 receptor, is an important mediator of the symptoms of allergies. H1-antihistamines are the treatment of choice for some chronic allergic conditions 30. Recently, it has been reported that blocking H1/H2 histamine receptors inhibits human cholangiocarcinoma tumorigenesis 31. Histamine H1 and H2 receptor signaling pathways play an important role in inflammation-related colon tumorigenesis 32. In addition, histamine improves the antitumor efficacy of PD-1/PD-L1 checkpoint blockade 33 and antihistamine drug ebastine inhibits cancer growth by targeting polycomb group protein EZH2 49. However, the antitumor effects of histamine H1 and H2 receptors in osteosarcoma have not been reported.

We first detected the antitumor properties of ebastine on osteosarcoma *in vivo* and *in vitro*. *In vitro*, the results illustrated that ebastine inhibited the proliferation and metastasis of osteosarcoma. Additionally, flow cytometry assays demonstrated that ebastine induced cell cycle arrest in S phase and promoted the apoptosis of osteosarcoma cells. In the subcutaneous tumor model, the size and weight of tumors were significantly decreased in response to treatment with ebastine. Simultaneously, in the lung metastasis model, there was a significant decrease in lung metastases in the ebastine group. Moreover, there were no obvious toxic effects of ebastine in the animal experiments. Furthermore, the results showed that ebastine reduced the protein expression of CDK2 and CyclinA2 and activated the expression of cleaved caspase-8 and -9. Moreover, *in vivo*, the results of western blotting and IHC assays were similar to the *in vitro* results. Taken together, these results demonstrated that ebastine inhibited proliferation and metastasis and promoted the apoptosis of osteosarcoma *in vitro* and *in vivo*.

To further study the antitumor activity of ebastine, we examined autophagy after treatment with ebastine and found that ebastine activated autophagy in osteosarcoma cells. Autophagy is a complex process regulated by the coordinated action of more than 30 autophagy-related proteins (ATGs) 34-35. Interest in autophagy in various fields has never diminished. Numerous studies have demonstrated that autophagy is used by tumor cells as a highly dynamic mechanism to repress the initial steps in carcinogenesis 36-38. Reports have shown that autophagy initiation is mediated by the ULK1 complex, which is regulated by AMP-activated protein kinase (AMPK) activation and inhibition of mammalian target of rapamycin (mTOR) 39-40. In this study, it was demonstrated that ebastine promoted autophagy by upregulating the protein expression of ATG7, ATG16 and LC3B in osteosarcoma cells. TEM directly revealed autophagosomes, and mRFP-GFP-LC3 fluorescence analysis indicated that ebastine mediated autophagic flux in osteosarcoma cells. Meanwhile, expression of LC3B was upregulated *in vivo*. After treatment with an autophagy inhibitor (3-MA), the results showed that 3-MA partially rescued ebastine-induced cell death and autophagy. All these results indicated that ebastine induced proapoptotic autophagy.

Furthermore, RNA-seq analysis was used to explore the underlying mechanism of the antitumor effect of ebastine. KEGG pathway analysis revealed that the AMPK signaling pathway was enriched in response to treatment with ebastine. Moreover, we revealed that ebastine activated the AMPK/ULK1 pathway by significantly increasing the expression of p-AMPK and ULK1 both *in vitro* and *in vivo*. The AMPK signaling pathway plays a major role in autophagy induction 41-42. Mihwa Kim reports that SRC phosphorylation leads to an increase in AMPK phosphorylation and LC3-II production in MG-63 cells 43. The selective inhibitor compound C (dorsomorphin) of AMPK was used to treat osteosarcoma cells. The results showed that dorsomorphin partially recovered apoptosis and led to decreased LC3-II production. All of these findings indicated that ebastine exerted antitumor activity through the AMPK/ULK1 signaling pathway.

To further determine how ebastine regulates the AMPK/ULK1 pathway in osteosarcoma, we analyzed the RNA-seq data and searched relevant reports. Twenty-one differentially expressed genes in the RNA-seq data were found to be related to autophagy in PubMed. First, we examined the expression of 21 genes after treatment with ebastine in three osteosarcoma cell lines. Ten genes were simultaneously upregulated in three osteosarcoma cell lines. Next, we knocked down the 10 genes via independent siRNAs and found that knockdown of IPMK decreased the expression of LC3B and p-AMPK. The interaction between IPMK and AMPK has been reported previously 44-45. IPMK mediates AMPK-dependent autophagy, and AMPK initiates autophagy by regulating the transcription of autophagic genes 46. In liver inflammation, IPMK-AMPK-SIRT1 and IPMK-AMPK-ULK1 appear to mediate the influence of IPMK on autophagy 47. Furthermore, deletion of IPMK markedly reduces the transcription of autophagy-associated genes and decreases activation of ULK as well as downstream autophagy signaling 48. In the present study, autophagic flux and western blot analysis demonstrated that knockdown of IPMK decreased autophagy in osteosarcoma cells compared to the ebastine-treated group. Therefore, the rescue assay revealed that induction of autophagy and the AMPK/ULK1 signaling pathway by ebastine treatment were reversed by knockdown of IPMK, indicating that the activity of ebastine was IPMK dependent.

## Conclusions

These results indicated that ebastine (a H1-histamine receptor antagonist) exerts antitumor activity in osteosarcoma. Ebastine exerts an antitumor effect and promotes autophagy by activating the AMPK/ULK1 signaling pathway, which is IPMK dependent. Our results provide insight into the clinical application potential of ebastine and indicate that ebastine might be a novel candidate to treat osteosarcoma patients.

## Supplementary Material

Supplementary figures and table.

## Abbreviations

AMPK: Adenosine 5'-monophosphate (AMP)-activated protein kinase
ATG7: Autophagy related protein7
ATG16: Autophagy related protein 16
AVs: autophagic vacuoles
BCA: the bicinchoninic acid
BECN1: Bclin1
CCK-8: Cell Counting Kit 8
CDK2: cyclin-dependent kinase 2
Co-IP: Coimmunoprecipitation
DAPI: 4′,6-diamidino-2-phenylindole
ECL: enhanced chemiluminescence
GFP: green fluorescent protein
HE: hematoxylin eosin
IC50: the half-maximal inhibitory concentration
IHC: immunohistochemistry
IPMK: Inositol polyphosphate multikinase
KEGG: Kyoto Encyclopedia of Genes and Genomes
LC3: microtubule-associated proteins 1A/1B
3-MA: 3-methyladenine
p-AMPK: Phospho AMP-activated protein kinase
PVDF: polyvinylidene difluoride
QRT-PCR: quantitative real-time polymerase chain reaction
FP: red fluorescent protein
RIPA: radio Immunoprecipitation Assay
SPF: specific-pathogen-free
TEM: Transmission electronic microscopy
TLC: thin layer chromatography
ULK1: unc-51-like kinase 1
