# Supplementary material for: Ebastine exerts antitumor activity and induces autophagy by activating AMPK/ULK1 signaling in an IPMK-dependent manner in osteosarcoma
Source: Int J Biol Sci. 2023 Jan 1;19(2):537–51. doi: 10.7150/ijbs.69541 (PMC9830506; doi:10.7150/ijbs.69541)
Supplement: Supplementary file 1 — Supplementary figures and table. [file ijbsv19p0537s1.pdf]

Supplementary Tables

Supplementary Table 1. The primer sequences for qRT-PCR

Gene name		Sequence (5'-3')
	Forward	CTTCCACCAGCGGAACTACC
FOSB	Reverse	ACTGATCTGTCTCCGTCTCCT
	Forward	GGACGGACCAAGCAGAGTTT
FSD2	Reverse	GGACGGACCAAGCAGAGTTT
	Forward	AGGGGTTCCTGGTCATACAC
LCP1	Reverse	TCCATTGCCATCAGTATCAACT
	Forward	GGACTGTCCTTTCGTGGCTC
HMGCS1	Reverse	GGCATGGTGAAAGAGCTGTG
	Forward	TGCACCATGGGAAGAAAGAAGA
PLA2G4C	Reverse	TACGTGACGGCATCCAACAG
	Forward	AGCTCTGTGTGAAGGTGCAG
CXCL8	Reverse	TCTCAGCCCTCTTCAAAAACTTC
	Forward	GTTCCGAGGTTGGAACACCT
MSMO1	Reverse	TCTCTGGCTTATCCTGAACGG
	Forward	GCTGTTCCCTGCGAAAGGTAT
IDI1	Reverse	TGTTGCTTGTCGAGGTGGTT
	Forward	TGGCATGCATGTAGCAGTGA
CLK4	Reverse	GCATCTGGACACATCGGAAGA
	Forward	TCTGGGCAGCAGGTTTACAA
BIRC3	Reverse	CCCGAGATTAGACTAAGTCCCTT
	Forward	TGCAGGAGGGTTCAAATCCG
N4BP3	Reverse	AGGCAGCTGCTTCATGGTG
	Forward	AGGTGGGTGAGGAAATCCAGA
AGR2	Reverse	TCATCAAGGGTTTGTTGCTTGT
	Forward	GGAACCTGAGGAGAGAGTGTTC
DDIT3	Reverse	CTGCCATCTCTGCAGTTGGA
	Forward	ACCCTTACAGGTAAGAATGGATCG
CPEB2	Reverse	ACCTCGTCTTCGCCCATAAC
	Forward	GAGAGAACATTGAGGCCCCC
MYBPH	Reverse	TTGCAGGTTGACCGTCTCTC
	Forward	GTGTGGATAGAGCACGGCTG
CHRND	Reverse	CTGCCAGTCGAAGGGGAAAT
	Forward	CAATGGAAACCCCCTCACGA
ZFHX2	Reverse	GTGACAGCCAGTACCACCTC
	Forward	CCTGATCTCTCCAAGTCCGC
PRR15	Reverse	CAGCGGCCTGCTCGG
	Forward	ATCGTGTGCACCTTCACCTA
KCNK3	Reverse	GTGCCCGTAGCCGATG
	Forward	CCATGGGACATCCCAGGTTTT
EPN3	Reverse	TTCCTGAGGGGATCGGAGAC

	Forward	GTCATGGACCTGGGTATGC
CCR7	Reverse	TCCACTGTGGTGTTGTCTCC
	Forward	CCGGCCGGGCTCTCAT
SLC1A7	Reverse	TCCGGACATCAAGCTGGAGA
	Forward	GCAGAGCGGCCAGCAA
LSP1	Reverse	GCTTCAGGCTGAGGAGCATC
	Forward	TGCAACACCCCTACAAGTGC
ADAMTSL2	Reverse	GTTGGGCTGTTGTCCGTGG
	Forward	ACGGACATGCAAGGTGTAGG
ALPK2	Reverse	TTCTTTTCGCCTGGGGTCTC
	Forward	AGAGATTGAGACTGCGTGGC
EIF2AK3	Reverse	AGAGATTGAGACTGCGTGGC
	Forward	ACTCCTGCGACCAGGTACT
PER1	Reverse	GCCATGGGGAGAACAGAACA
	Forward	GCTCCGAGGAACTTTCTCCC
KLF6	Reverse	GCTCCGAGGAACTTTCTCCC
	Forward	AGCCATGATCAGGCATTGGT
GBE1	Reverse	GTAGCGAAGAAGGTCGTCGT
	Forward	GTTTCCGTCGCCCTGATGTA
ADM	Reverse	GCATCCGGACTGCTGTCTT
	Forward	GTACGAGTCGGCCAAGTTGA
GADD45B	Reverse	GTGTGAGGGTTCGTGACCA
	Forward	GGGACTTGCTTCCAAAGGAAAA
HERPUD1	Reverse	CCTTGGCGTTGATTTCTGGC
	Forward	CAGCCAGGTCGGCAGTATAG
JUN	Reverse	GGACTCTGCCACTTGTCTCC
	Forward	AGGCCCCGCCTCTAGTTC
ACSS2	Reverse	AGGCCCCGCCTCTAGTTC
	Forward	TCGCCCTGGAGTAATTTCGG
NOG	Reverse	CTGGGTGTTCGATGAGGTCC
	Forward	CATCAACGAGCCTACGGCA
HSPA5	Reverse	TCCATGACACGCTGGTCAAA
	Forward	CTCCTCCTCGAAAGATGGCT
HDAC9	Reverse	TTCCACATGAGGTCAGGCTGT
	Forward	CTTGTCTCTGCAGACCGCT
NR1D1	Reverse	CTTGTCTCTGCAGACCGCT
	Forward	CAGGATGAAATTTGGCATGGGG
BBC3	Reverse	CAGGATGAAATTTGGCATGGGG
	Forward	GCGCACTAGAACGAGCAAG
TRAF6	Reverse	GCGCACTAGAACGAGCAAG
	Forward	ACTGCAACATCCAGATGGCA
IPMK	Reverse	ACTGCAACATCCAGATGGCA
	Forward	AGAAAACCGTGGATGGAATTAGAA
CHMP2B	Reverse	AGAAAACCGTGGATGGAATTAGAA

KLF4	Forward	GGTCAGTCCCGGGGATTTGT
	Reverse	CAGTGGTAAGGTTTCTCACCTGTGT
HSPA1A	Forward	ACATCAGCCAGAACAAGCGA
	Reverse	ACATCAGCCAGAACAAGCGA
PPP1R15A	Forward	CTAAAGGCCAGAAAGGTGCG
	Reverse	ACGAAGGGACAGAGGAGGAA
PIM1	Forward	CTGGGGAGAGCTGCCTAATG
	Reverse	GCTCCCCTTTCCGTGATGAA
β-actin	Forward	GTCATTCCAAATATGAGATGCGT
	Reverse	GCATTACATAATTTACACGAAAGCA

Supplementary Figures and figure legends:

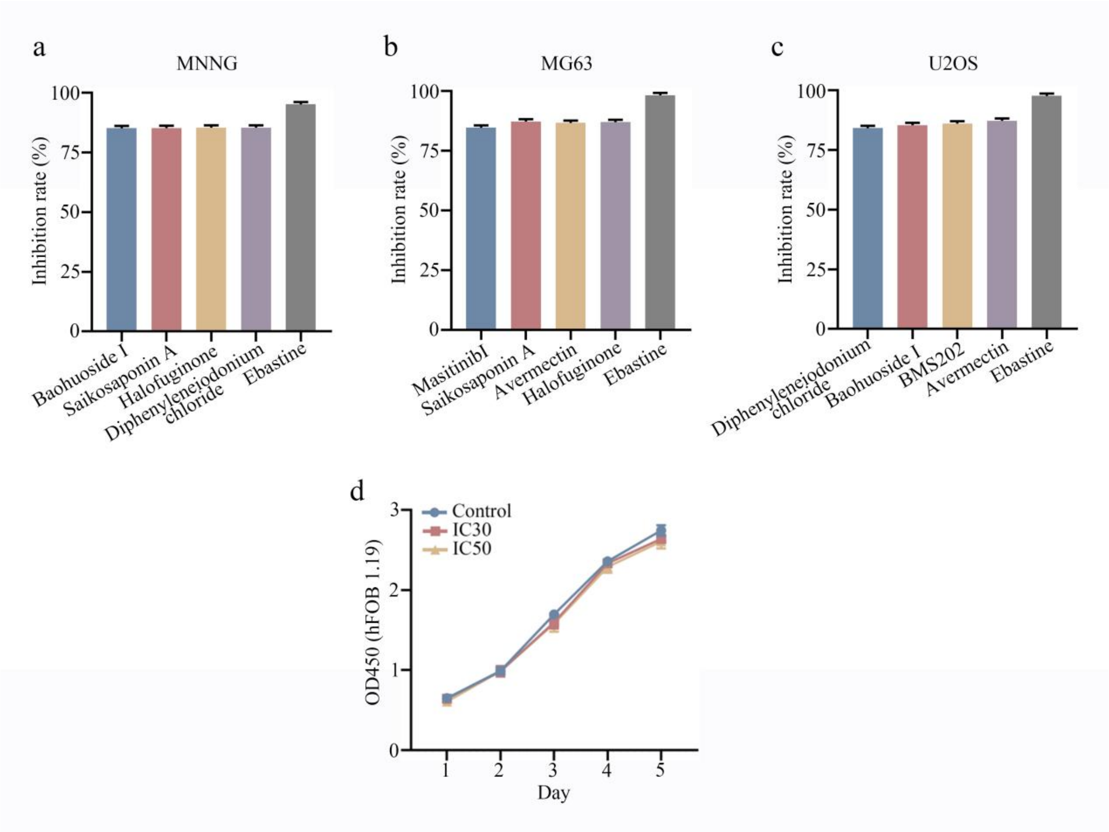

Supplementary Figure 1. (a-c) The inhibition of the first 5 effective drug in all three osteosarcoma cell lines. (d) CCK-8 assay was used to detect the proliferation of osteoblast cell hFOB 1.19 after treatment with the IC30 and IC50 of ebastine.

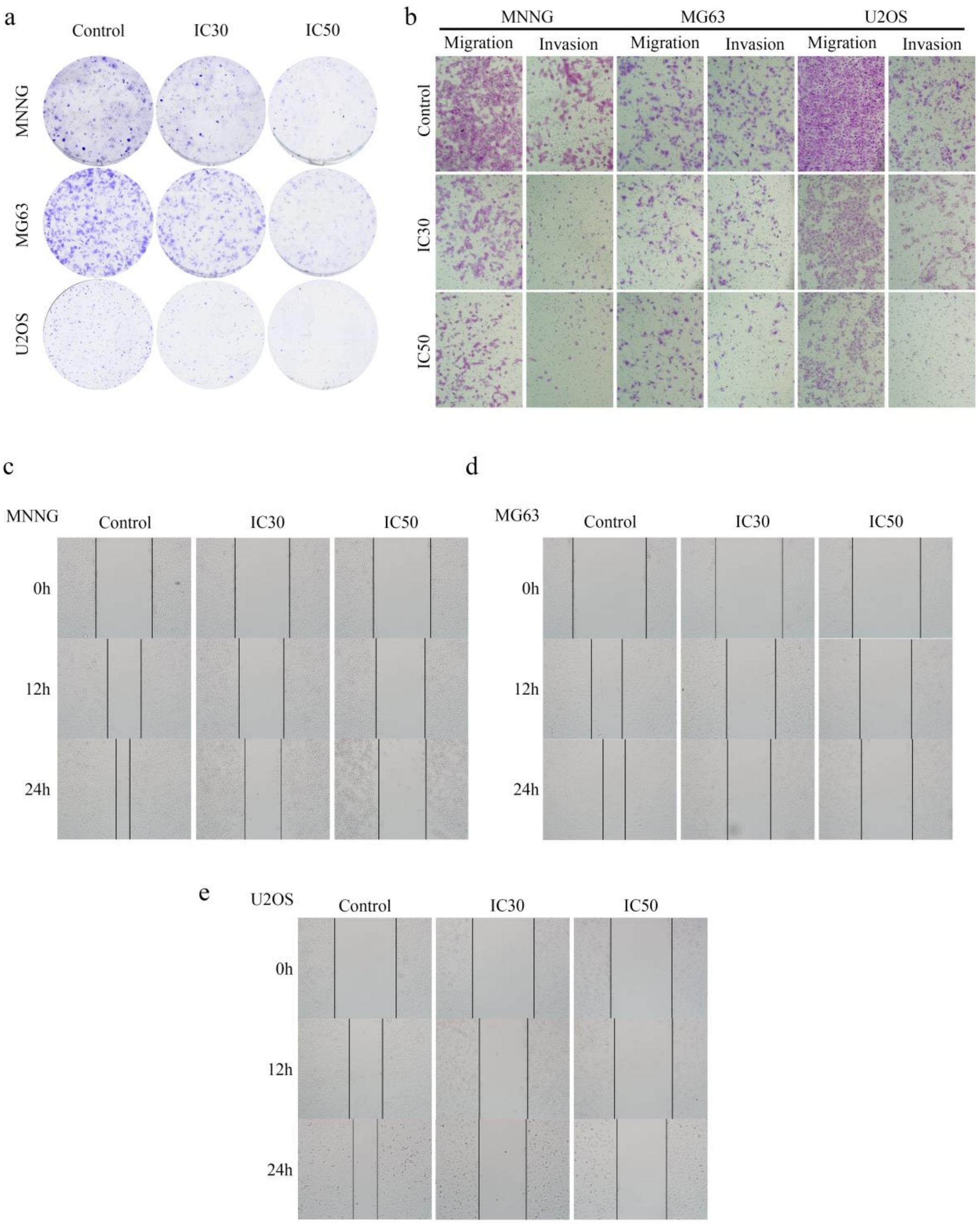

Supplementary Figure 2. (a) Colony formation assays in osteosarcoma cells after treatment with the IC30 and IC50 of ebastine (magnification, ×40). (b) Transwell assays in osteosarcoam cells after treatment with the IC30 and IC50 of ebastine (magnification, ×200). (c-e) A wound-healing assay was used to determine cell migration after treatment with the IC30 and IC50 of ebastine (magnification, ×200). Data are shown as the means ± SD from at least three independent experiments. Statistical analysis was performed using Student's t-test. Error bars represent the SEM. $*P<0.05$; $** P<0.01$; $*** P<0.001$.

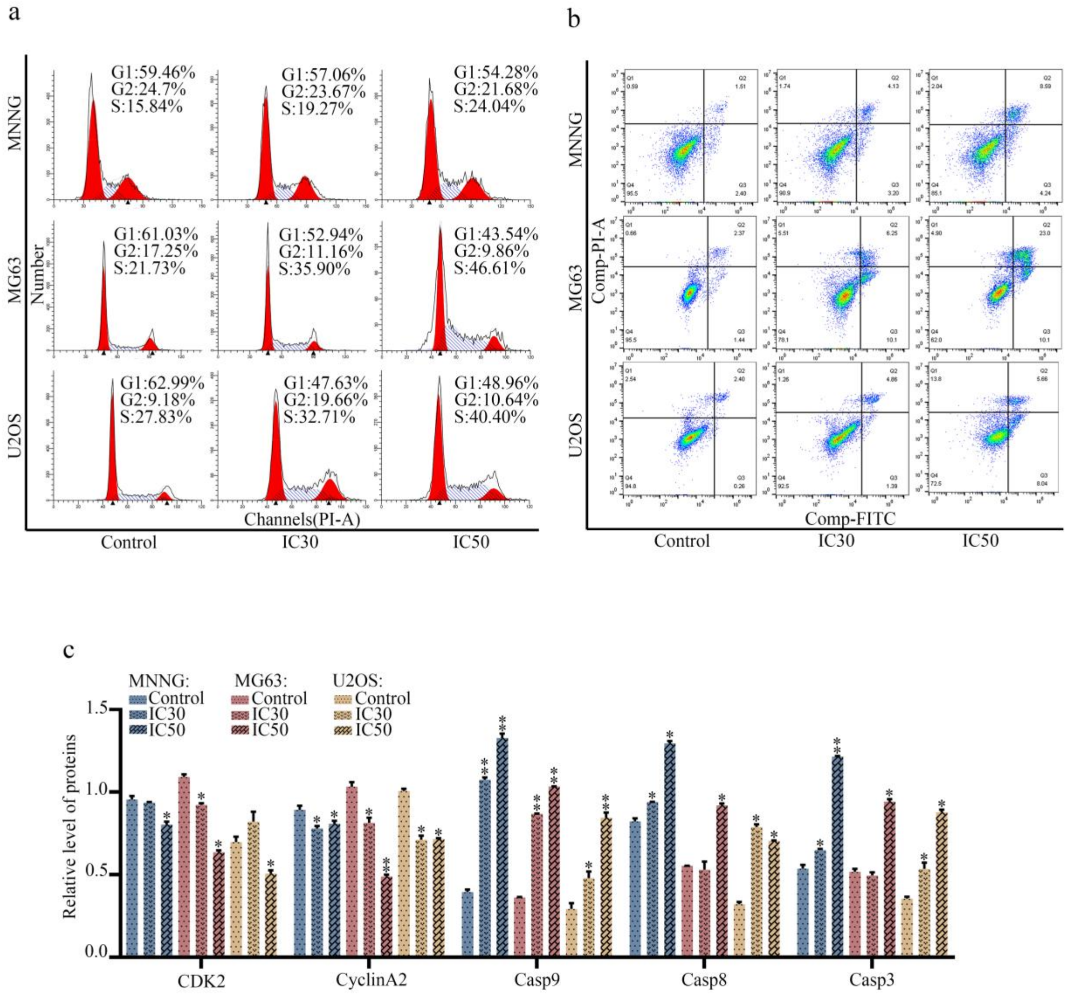

Supplementary Figure 3. (a) Flow cytometry of cell cycle distribution in each phase after treatment with the IC30 and IC50 of ebastine after 48 h. (b) Flow cytometry of cell apoptosis distribution after treatment with the IC30 and IC50 of ebastine for 48 h. (c) Protein levels of cell cycle- and apoptosis-related proteins after treatment with ebastine for 48 h, assayed by Western blot. Statistical analysis was performed using Student's t-test. Error bars represent the SEM. *$P<0.05$; ** $P<0.01$.

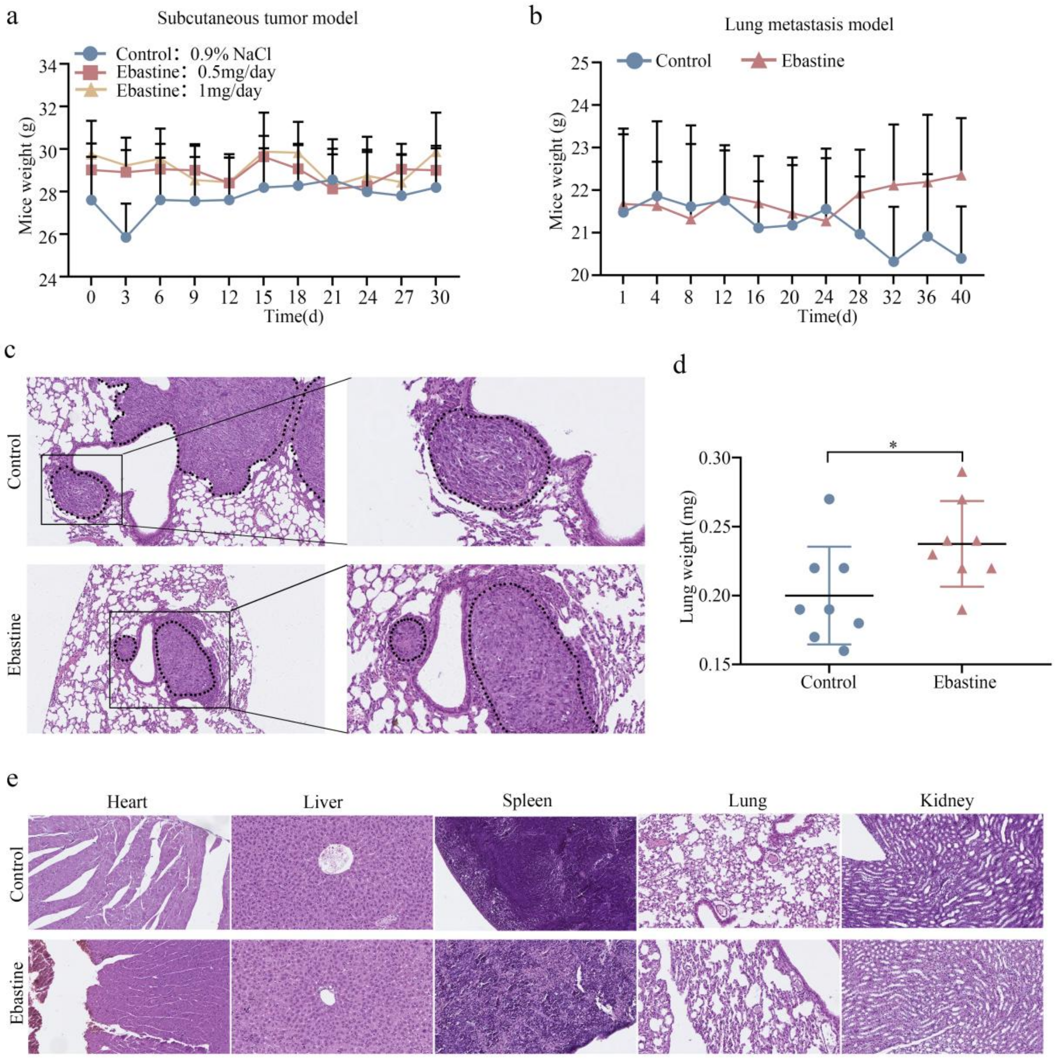

Supplementary Figure 4. (a-b) Growth curve drawn by measuring the weight of mice on the indicated days in subcutaneous tumors and lung metastasis model. (c) HE staining of lung samples obtained from nude mice after injection with MNNG cells. (d) Diagram showing lung weight after treatment with ebastine. (e) HE staining of the heart, liver, spleen, lung, and kidney organs in ebastine-treated MNNG mice and control mice. Data are shown as the means ± SD from at least three independent experiments. Statistical analysis was performed using Student's t-test. Error bars represent the SEM. *P<0.05.

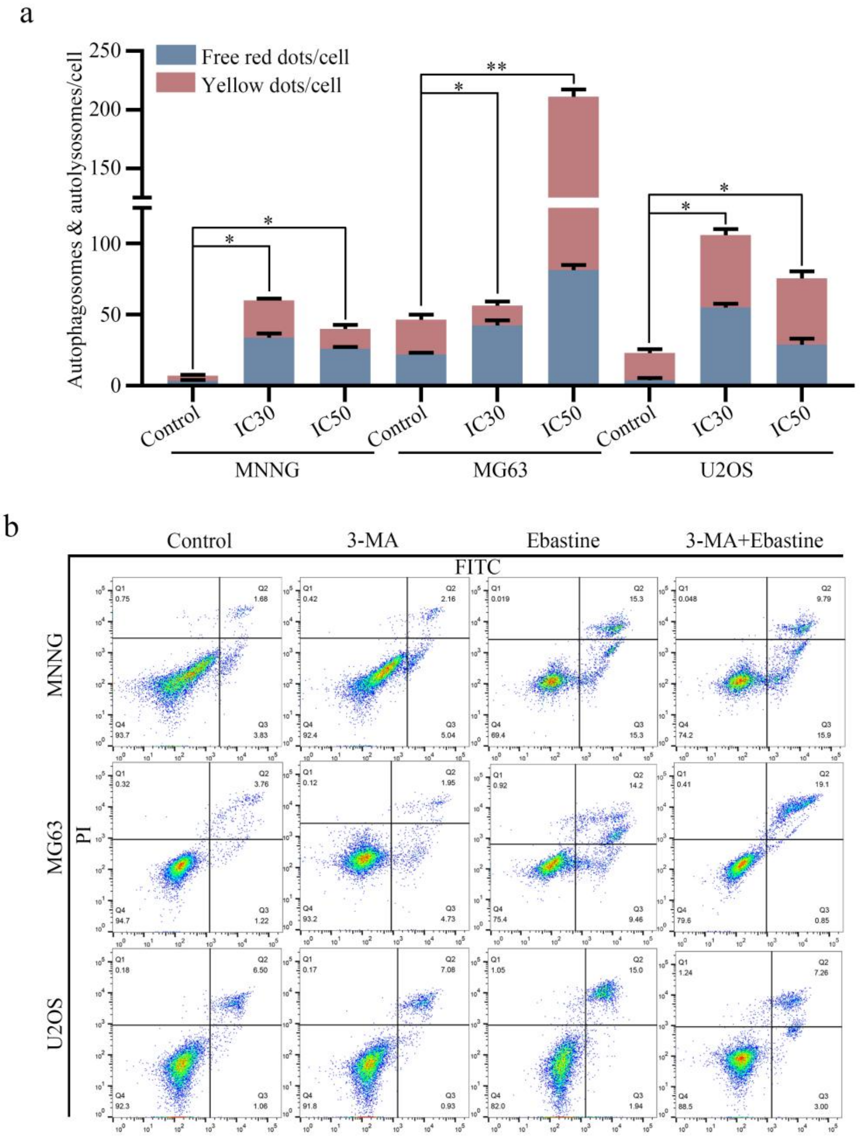

Supplementary Figure 5. (a) LC3 puncta were analyzed using the mRFP-GFP-LC3 construct after treatment with ebastine for 48 h. (b) Flow cytometry was used to analyze changes in apoptosis after treatment with ebastine for 48 h with or without 3-MA. Data are shown as the means ± SD from at least three independent experiments. Statistical analysis was performed using Student's t-test. Error bars represent the SEM. **P*<0.05; ** *P*<0.01.

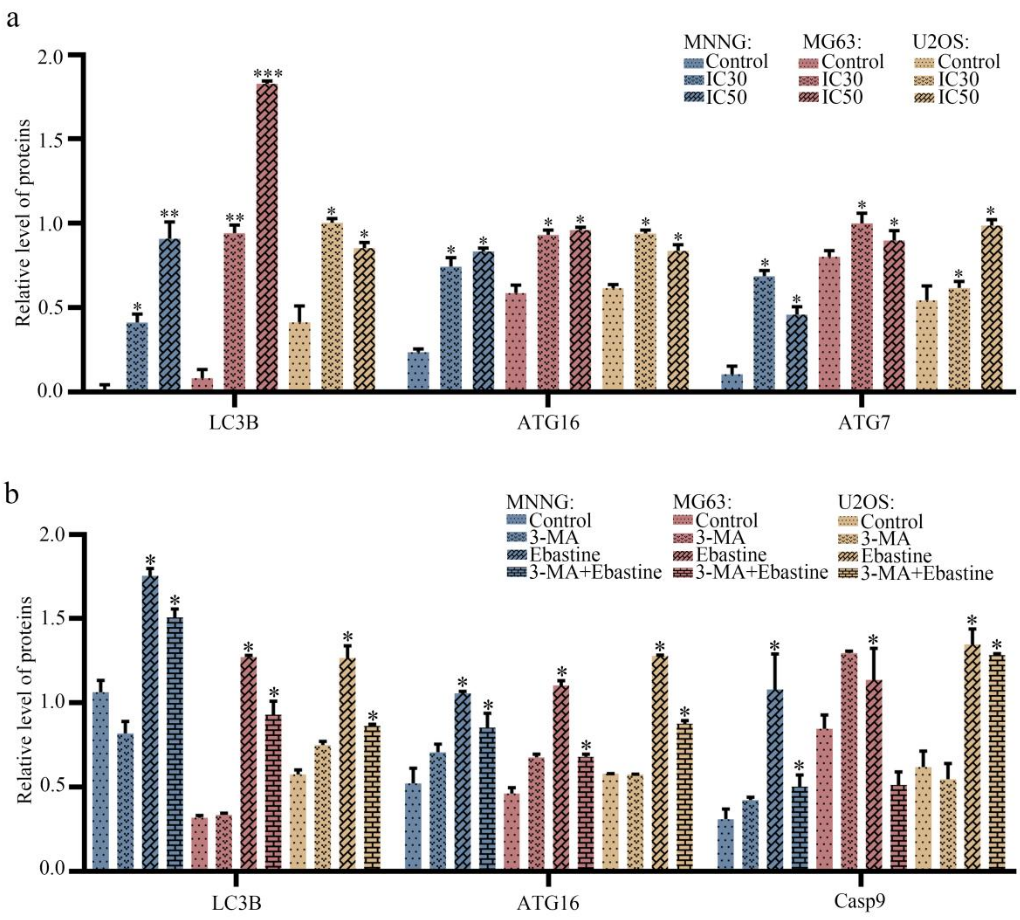

Supplementary Figure 6. (a) Protein levels of LC3B, ATG16 and ATG7 after treatment with the IC30 and IC50 of ebastine for 48 h, assayed by Western blot. (b) Autophagy-related proteins were analyzed by Western blot after treatment with ebastine for 48 h with or without 3-MA. Data are shown as the means ± SD from at least three independent experiments. Statistical analysis was performed using Student's t-test. Error bars represent the SEM. *P<0.05; ** P<0.01; *** P<0.001.

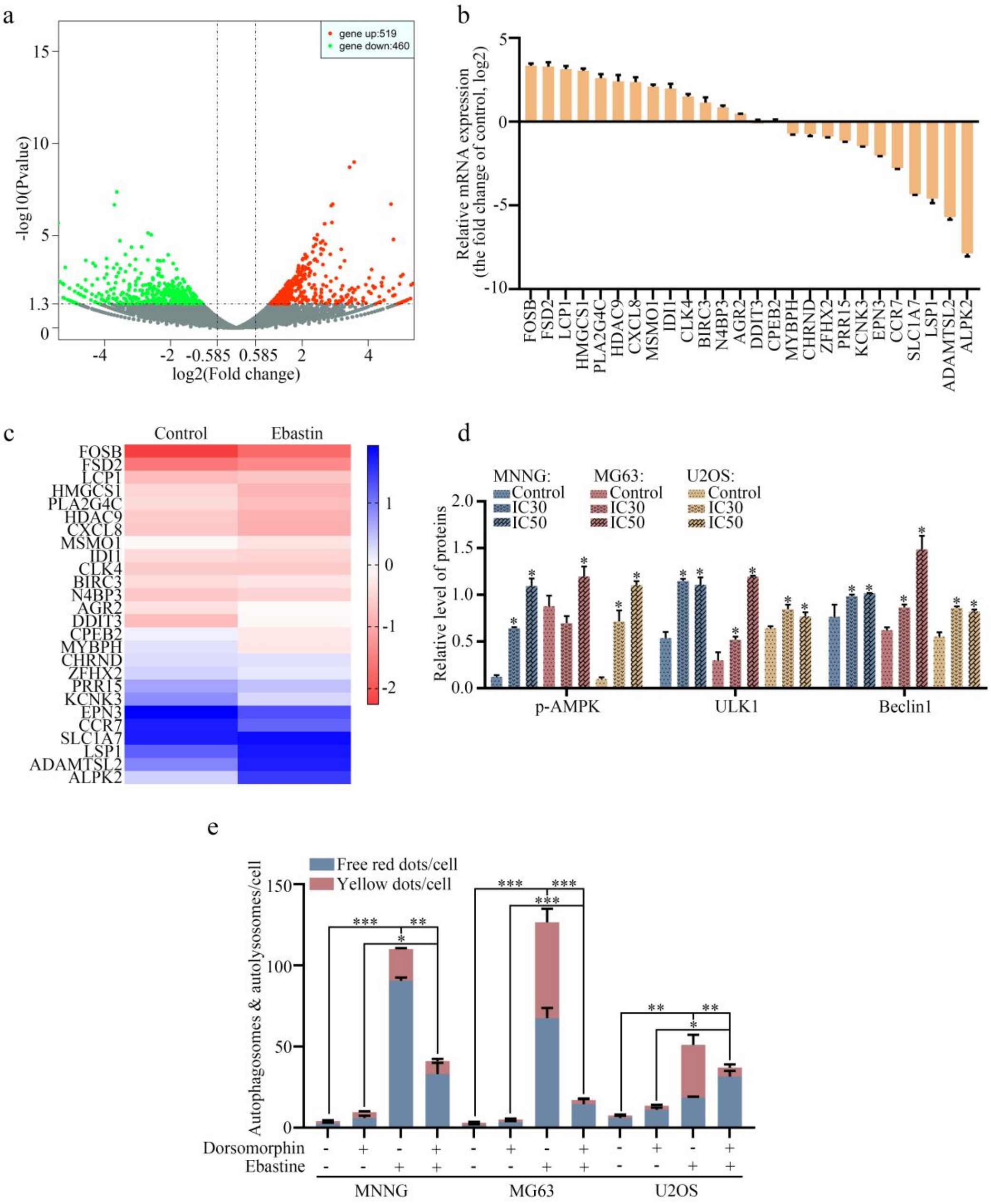

Supplementary Figure 7. (a) A volcano plot analysis revealed 519 up-regulated and 460 downregulated genes. (b) The mRNA expression levels of 15 up-regulated and 11 down-regulated genes in osteosarcoma cells. (c) Heatmap displaying the mRNA expression levels of 15 up-regulated and 11 down-regulated genes in osteosarcoma cells. (d) Western blot showing the protein expression of p-MEK1 and LC3B after treatment with ebastine. (e) LC3 puncta were analyzed by the mRFP-GFP-LC3 construct after treatment with ebastine for 48 h with or without dorsomorphin. Data are shown as the means ± SD from at least three independent experiments. Statistical analysis was performed using Student's t-test. Error bars represent the SEM. *$P<0.05$; ** $P<0.01$; *** $P<0.001$.

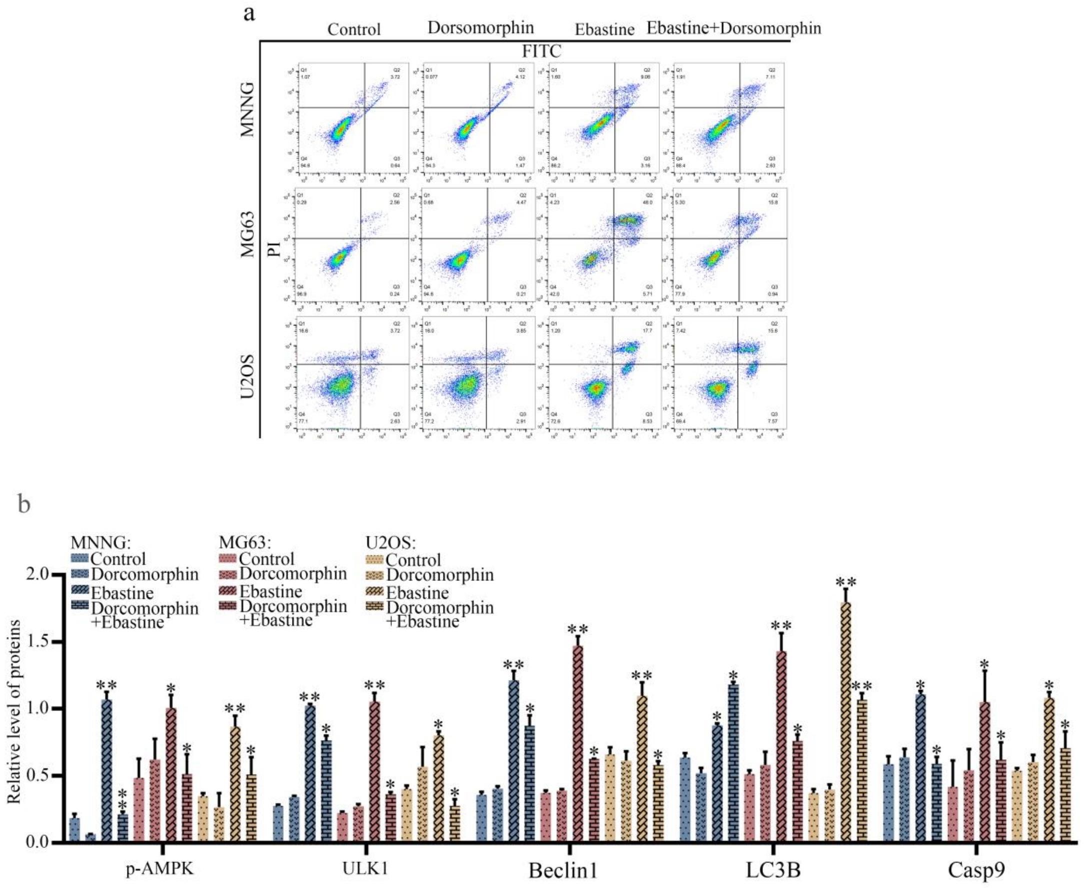

Supplementary Figure 8. (a) Flow cytometry was used to analyze changes in apoptosis after treatment with ebastine for 48 h with or without dorsomorphin. (b) The relative protein levels of p-AMPK, ULK1, Beclin1, LC3B and Casp9 after treatment with ebastine for 48 h with or without dorsomophin. Data are shown as the means ± SD from at least three independent experiments. Statistical analysis was performed using Student's t-test. Error bars represent the SEM. *P<0.05; ** P<0.01.

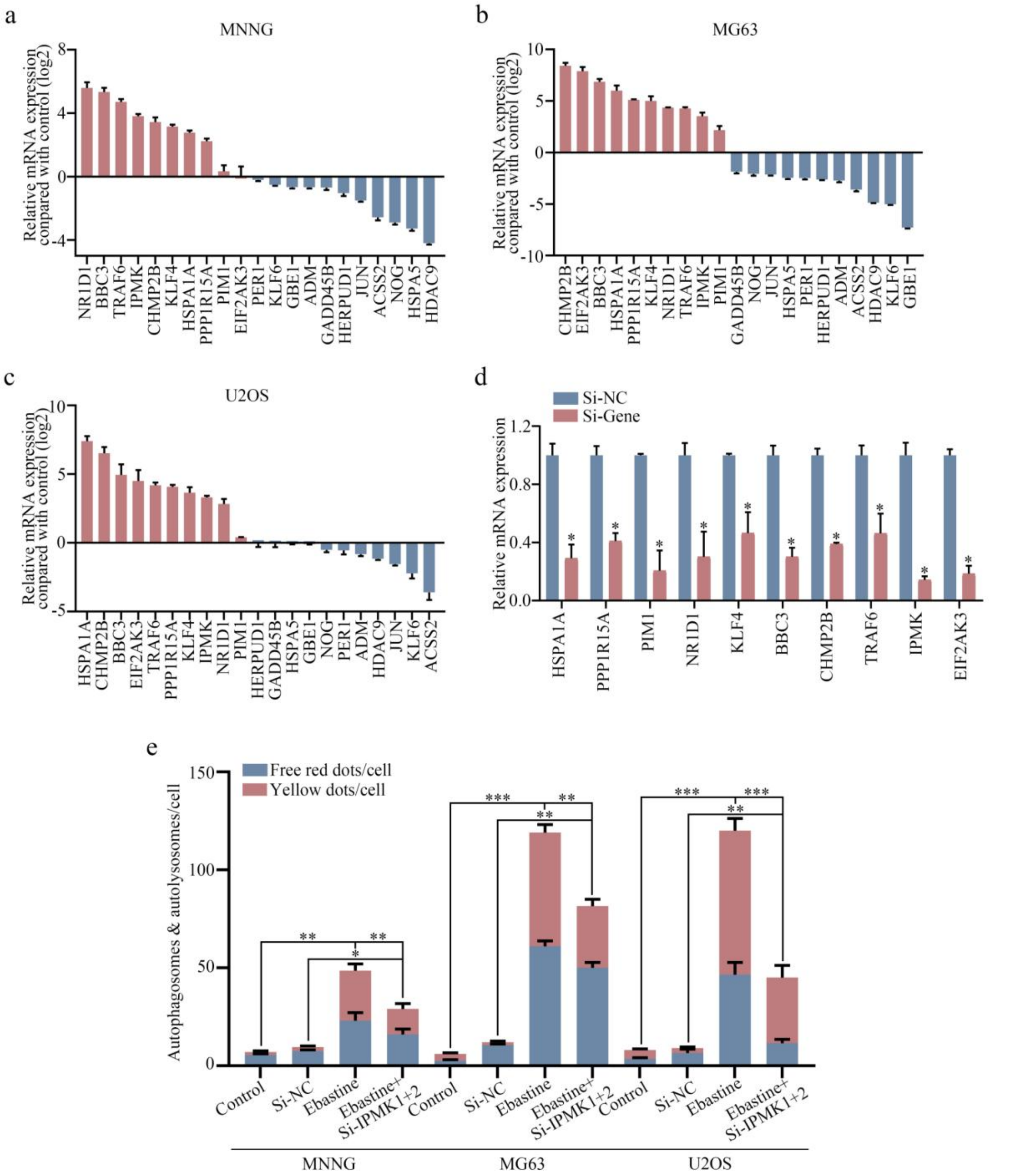

Supplementary Figure 9. (a-c) The mRNA expression of 21 genes after treatment with ebastine in three osteosarcoma cell lines. (d) The relative mRNA expression of the 10 genes was determined by qRT-PCR after transfection with independent siRNAs in MNNG cells. (e) LC3 puncta were analyzed by the mRFP-GFP-LC3 construct after treatment with ebastine for 48 h with or without knockdown of IPMK. Data are shown as the means ± SD from at least three independent experiments. Statistical analysis was performed using Student's t-test. Error bars represent the SEM. **P*<0.05; ** *P*<0.01; *** *P*<0.001.

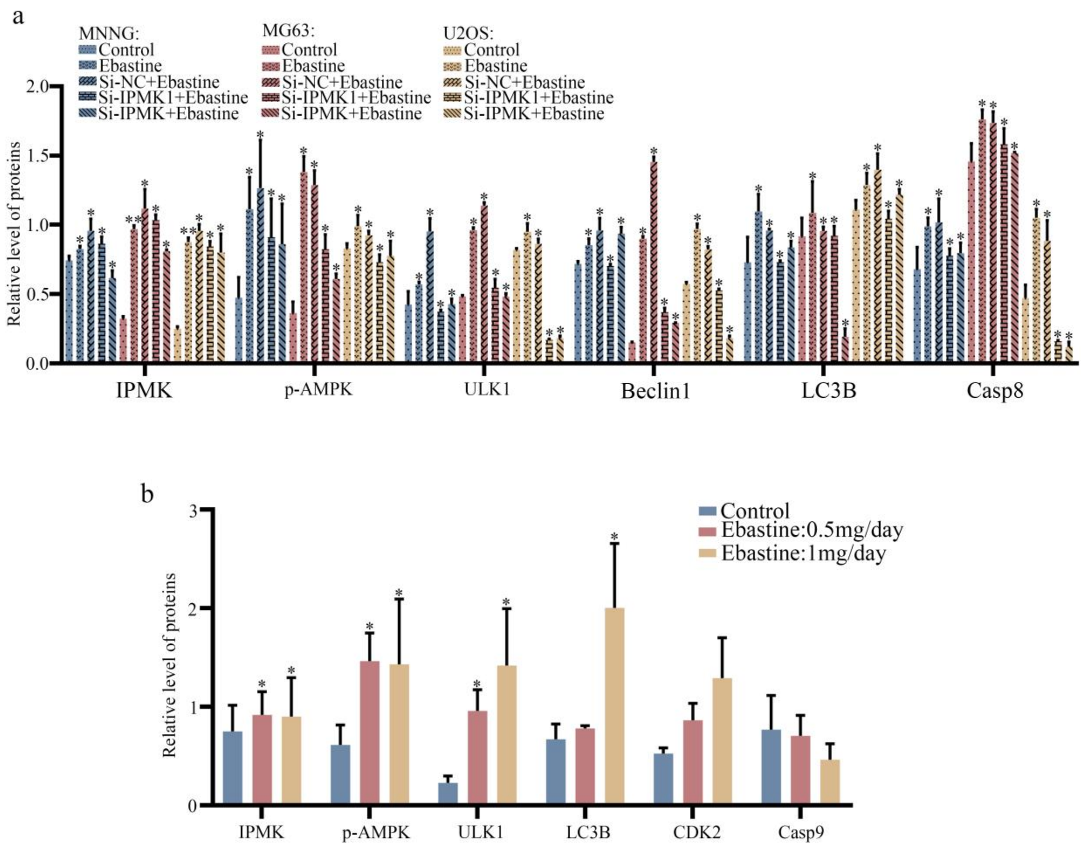

Supplementary Figure 10. (a) Protein levels of apoptosis, autophagy, IPMK, and p-AMPKK in osteosarcoma cells treated with ebastine for 48 h with or without knockdown of IPMK. (b) Protein expression of IPMK, p-AMPK, ULK1, LC3B, and CDK2 and cleavage of caspase-9 were analyzed by western blot in tumor tissues. Statistical analysis was performed using Student's t-test. Error bars represent the SEM. **P*<0.05.

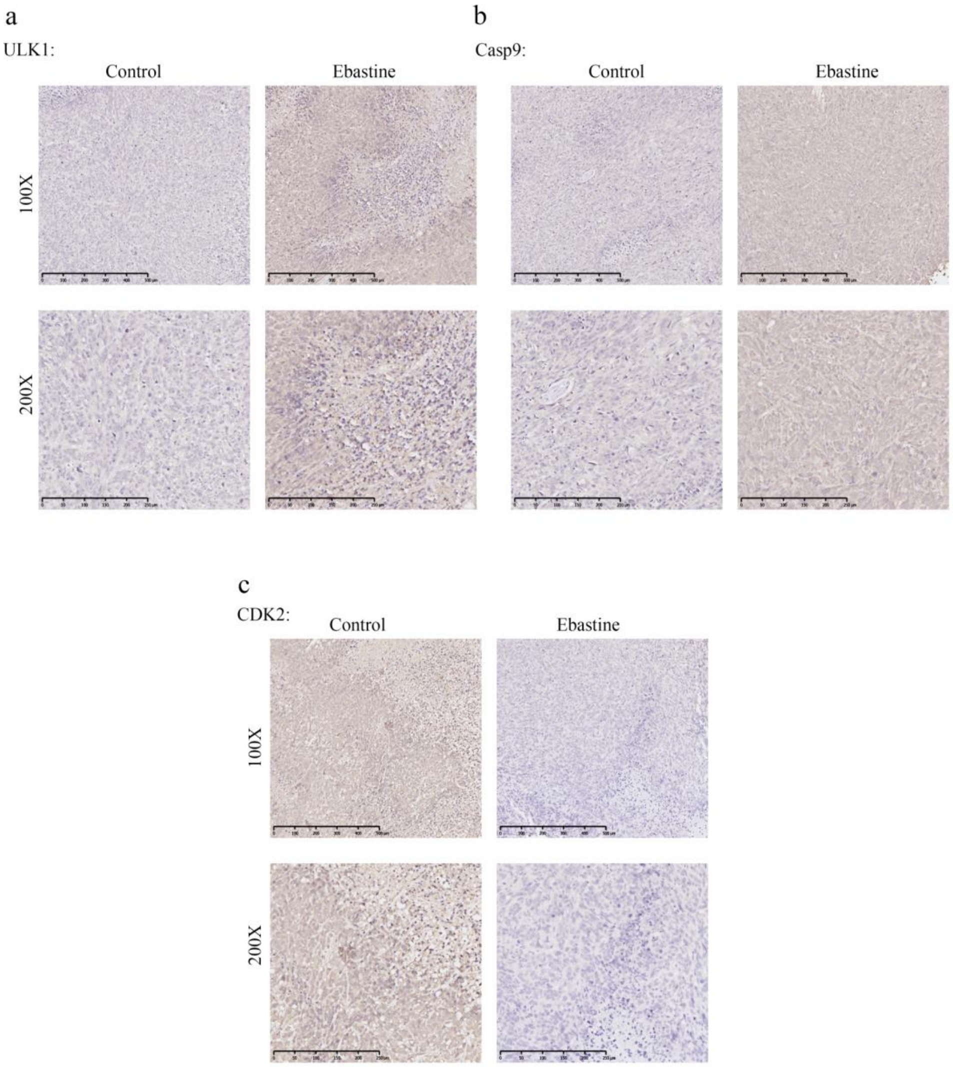

Supplementary Figure 11. (a-c) IHC analysis of the related proteins ULK1, CDK2, and caspase-9.
